# The promotive and protective effects of parents’ perceived changes during the COVID-19 pandemic on emotional well-being among U.S. households with young children: an investigation of family resilience processes

**DOI:** 10.3389/fpsyg.2023.1270514

**Published:** 2024-01-08

**Authors:** Sihong Liu, Stephanie M. Curenton, Jacqueline Sims, Philip A. Fisher

**Affiliations:** ^1^Stanford Center on Early Childhood, Stanford University, Stanford, CA, United States; ^2^Center on Ecology of Early Development, Boston University, Boston, MA, United States

**Keywords:** perceived positive changes, COVID-19 pandemic, early childhood development, material hardship, economic instability, family resilience, promotive and protective factors

## Abstract

**Introduction:**

The COVID-19 pandemic may constitute a traumatic event for families with young children due to its acute onset, the unpredictable and ubiquitous nature, and the highly distressing disruptions it caused in family lives. Despite the prevalent challenges such as material hardships, child care disruptions, and social isolation, some families evinced remarkable resilience in the face of this potentially traumatic event. This study examined domains of changes perceived by parents of young children that were consistent with the post-traumatic growth (PTG) model as factors that facilitate family resilience processes.

**Methods:**

This study drew data from the RAPID project, a large ongoing national study that used frequent online surveys to examine the pandemic impact on U.S. households with young children. A subsample of 669 families was leveraged for the current investigation, including 8.07% Black, 9.57% Latino(a), 74.44% non-Latino(a) White families, and 7.92% households of other racial/ethnic backgrounds. In this subsample, 26.36% were below 200% federal poverty level.

**Results:**

Approximately half of the parents reported moderate-to-large degrees of changes during the pandemic, and the most prevalent domain of change was appreciation of life, followed by personal strengths, new possibilities, improved relationships, and spiritual growth. Black and Latino(a) parents reported more changes in all five domains than White parents and more spiritual growth than parents of the other racial/ethnic groups. Moreover, parent-reported improved relationships were found to indirectly reduce young children’s overall fussiness/defiance and fear/anxiety symptoms through reducing parents’ emotional distress. Perceived changes in the new possibilities, personal strengths, and appreciation of life domains were found to serve as protective factors that buffered the indirect impacts of material hardship mean levels on child behavioral symptoms via mitigating parents’ emotional distress.

**Discussion:**

These findings shed light on resilience processes of a family system in a large-scale, disruptive, and stressful socio-historical event such as the COVID-19 pandemic. The five PTG domains could inform therapeutic and intervention practices in the face of future similar events. Importantly, these findings and the evinced family resilience should not negate the urgent needs of policy and program efforts to address material hardships, financial instabilities, and race/ethnicity-based structural inequalities for families of young children.

## 1 Introduction

The COVID-19 pandemic profoundly disrupted the lives of families with young children and induced high parenting stress. Many U.S. households of young children have experienced multifaceted challenges, such as severe material hardships (i.e., difficulty paying for basic needs; Liu et al., 2022), employment instabilities (Patrick and Pybus, 2022), limited healthcare access (Akseer et al., 2020), child care disruptions (Amadon et al., 2022), and social isolation (Lee et al., 2022). With the high prevalence of these pandemic challenges, the whole population of U.S. households of young children was severely impacted. However, certain subpopulations were impacted more than the others due to prolonged systemic inequities. Specifically, the pandemic was a “racial macro stressor” (Seaton, 2020), as persisting structural racism and discrimination exacerbated challenges facing families of color, especially Black and Latino(a) households, making them disproportionally and negatively affected by the pandemic compared to White households (Iruka et al., 2021); similarly, families with lower income levels faced disproportionally more severe challenges than higher-income ones because of socioeconomic-status (SES) inequities (Feinberg et al., 2022).

These unprecedented, ubiquitous challenges related to the COVID-19 pandemic posed substantial risks of worsened emotional well-being for both parents and young children (Prime et al., 2020), especially disproportionally affecting households of lower-SES or families of color due to their experiences of structural inequities. However, some parents have exhibited remarkable mental and emotional tenacity, determined to persist and persevere in difficult circumstances to support their families. Through such tenacity, parents may perceive positive changes in their own strengths and capabilities, relationships with others, and philosophy of life, *despite* the stressful, potentially traumatic stressors. These perceived areas of mental and emotional positive changes could facilitate processes of family resilience as shown in enhanced family relationships (Vaterlaus et al., 2021), maintained well-being (McArthur et al., 2023), and continued on-track developmental and learning progresses (Timmons et al., 2021). This study aimed to better understand the role of parents’ self-reported mental and emotional positive changes during the pandemic using a theoretical and empirical frame referred to as the post-traumatic growth (PTG) model (Tedeschi and Calhoun, 2004; Tedeschi et al., 2018). Although we proposed the PTG model as the framework for this study, it is important to note that choosing to describe parents’ perspectives on PTG was an attempt to describe families’ experiences from an asset-based lens by demonstrating how parents were able to describe their positive emotional and mental positive changes, despite being burdened by the pandemic that disproportionally affected families of color. Adopting this framework in no way attempted to diminish the lived hardships facing families with young children. Rather, we adopted the PTG framework to tell the stories where families, especially those of color, demonstrated remarkable tenacity in how they perceived this stressful event by self-reporting areas of positive change within their family, personal, and spiritual lives, which might have actually helped them weather the pandemic.

Adopting a systems approach is essential to a better understanding of the processes underlying risk and resilience in early childhood development. As Masten and Motti-Stefanidi (2020) discussed, the COVID-19 pandemic was a multi-system disaster that disrupted nearly all systems related to early childhood development (e.g., the family life, healthcare, early childhood education, and social services) and interfered with the interconnections and interactions among these systems (Yoshikawa et al., 2020). Relatedly, resilience in the pandemic context stems from successful adaptation of a dynamic—an individual, a family, or a community—to disruptions. This study particularly focused on the *risk and resilience processes in the family system*. Because of young children’s heightened sensitivity to early caregiving experiences (Fox et al., 2010), adversities and stressors in early experiences may be particularly detrimental, while successful adaptation of the family system to disruptions may play a key role in fostering healthy child development. The importance of the family system was further underscored during the pandemic, because young children spent extended time with their parents at home where ubiquitous pandemic stressors had perpetuated. Moreover, as suggested by the Family Disruption Model (FDM; Prime et al., 2020), family risk and resilience processes during the pandemic often manifest in a *cascading* fashion (Lengua et al., 2022): shifts in family dynamics due to pandemic social disruptions often first led to changes in parent well-being (Eales et al., 2021), which could subsequently indirectly affect young children’s behaviors and development (Araújo et al., 2021; Samji et al., 2022).

One major challenge facing households of young children during the pandemic is severe material hardships. During the 3 years of pandemic, between 25 to 50% of families with children under 6 years old had difficulty paying for very basic needs (RAPID Study, 2023), constituting a major source of family stress. There has been extensive, compelling evidence on the detrimental impact of material hardship levels on parents’ and young children’s emotional well-being (Zilanawala and Pilkauskas, 2012; Zalewski et al., 2023). In addition to the mean level of material hardships, emerging research also highlights the negative impact of economic *unpredictability*—week-to-week/month-to-month fluctuation in family income levels and hardship status—on well-being (Hill et al., 2013; Liu et al., 2022). Indeed, unpredictability has been increasingly acknowledged as a key dimension of adversity that fundamentally alters child neurobiological processes underlying developmental outcomes and behavioral adaptation (Ellis et al., 2022; Liu and Fisher, 2022). Beyond the perception about the family’s income and financial situations on an average level, the uncertainty about the family’s ability to afford basic needs in the upcoming weeks/months could also affect parents’ and children’s emotions and behaviors. Empirical evidence on the impacts of economic unpredictability on child development and the processes that could mitigate these impacts is urgently needed to advance our understanding about early experience unpredictability, and to inform intervening program and policy efforts. As such, our examination of pandemic disruptions focused on families’ material hardship experiences and incorporated both mean levels and unpredictability of hardship experiences.

The influences of material hardship experiences on family well-being may manifest via a *cascading effect* as suggested in the FDM (Prime et al., 2020), where hardship mean levels and unpredictability exert effects on child behaviors through disrupting the proximal caregiving processes. In particular, parents facing hardships may have higher stress levels, more family conflicts, and less energy to consistently, warmly interact with their children (Hill et al., 2013). With unstable income or uncertainty about their abilities to pay for basic needs, parents may also face challenges in financial management, decision-making, and routines disruptions (Liu et al., 2022). These factors could induce poorer mental health among parents of young children (Gershoff et al., 2007). In turn, children reared in such family environments may have difficulty meeting their socioemotional and cognitive developmental needs or developing effective self-regulation skills, which could further induce behavioral symptoms (Crespo et al., 2019; Glynn et al., 2021). Essentially, the effects of material hardships on family well-being could constitute a “chain of hardship,” where hardship mean levels and unpredictability indirectly increase child behavioral problems via elevated parent emotional distress (RAPID Study, 2020).

Despite the prevalence and severity of hardships facing households of young children, many parents and young children maintained their emotional well-being, signaling resilience. According to the FDM (Prime et al., 2020), intact or strengthened supportive factors in individual traits and/or family processes could have played a central role in either directly promoting better well-being outcomes of parents and young children (i.e., promotive effects) or buffering against pandemic negative effects (i.e., protective effects; Masten and Motti-Stefanidi, 2009). This model has received extensive empirical support in the first 2.5 years of the pandemic, with strong evidence on the effects of parent and family functioning on child adjustment (see Shoychet et al., 2023 for a systemic review). Aligned with the FDM, this study focused on investigating how parents’ perceived changes in the pandemic, which were derived from the post-traumatic growth (PTG) model and reflected positive parent functioning, could facilitate the processes of family resilience in the context of the COVID-19 pandemic. We specifically examined the direct promotive effects and the moderative protective effects of parents’ perceived changes during the pandemic across five domains—new possibilities, personal strengths, improved relationships, spiritual growth, and appreciation of life—on young children’s behavioral adaptation through the cascading processes involving parent emotional well-being. Notably, in this study, family resilience was theorized as the *processes* involving these perceived changes, parent well-being, and child adaptation; parents’ perceived changes were conceptualized as a parent functioning factor that could facilitate family resilience processes rather than a direct indicator of resilience.

Although the COVID-19 pandemic itself does not necessarily fit into the prevailing Post-Traumatic Stress Disorder (PTSD) models (Ehlers and Clark, 2000; Knox, 2003) or diagnostic criteria (American Psychiatric Association, 2013), pandemic stressors shared common characteristics with traumatic events, such as having acute onsets, being continuous and unpredictable, and being highly distressing (Bridgland et al., 2021). Recent studies have also found traumatic stress responses and PTSD-like symptoms among individuals who experienced the pandemic (Shevlin et al., 2020; Bridgland et al., 2021). As such, COVID-19 could be considered as a traumatic stressor event for some individuals, which had the capacity to elicit PTSD-like symptoms and intensify other mental health symptoms (Vazquez et al., 2021). Similar to our understanding of PTSD etiology, conceptualizing the pandemic as a traumatic stressor event does not mean that all individuals exposed to this event would inevitably develop relevant symptoms and suffer from its negative mental health consequences (Bonanno et al., 2011). Indeed, studies found that over half of individuals exposed to traumatic stressors may show post-traumatic positive changes as a result of perceived benefits or changes in self-perception, life philosophy, and interpersonal relationships (Helgeson et al., 2006; Tedeschi et al., 2018). Explanatory models of trauma have also begun incorporating this potentially positive aspect of traumatic experiences, suggesting that post-traumatic positive changes could be related to resilience processes (Anderson, 2018; Brooks et al., 2020).

The work by Tedeschi and Calhoun on PTG suggested three broad categories of potential changes after individuals’ facing and struggling with major difficulties, including perceived changes in self, a changed sense of relationships with others, and a changed philosophy of life (Tedeschi and Calhoun, 1996; Calhoun and Tedeschi, 1998; Calhoun et al., 2010; Tedeschi et al., 2018). Living through stressful and potentially traumatic events may provide individuals new information about their competence in coping with difficult situations, increase their sense of self-reliance and personal strength, and enhance their confidence to successfully handle future challenging situations (Tedeschi and Calhoun, 1996; Calhoun et al., 2010). The experiences of traumatic events may also prompt the individual to be more inclined to self-disclose and seek support, increasing their emotional expressiveness, willingness to accept help, and further strengthening their interpersonal relationships with others (Tedeschi and Calhoun, 1996; Calhoun et al., 2010). Moreover, traumatic experiences may facilitate the individual to reflect on the meaning and change the priorities in their lives, making them appreciate their life more, and in some cases, inducing changes in the domain of spiritual and existential matters (Tedeschi and Calhoun, 1996; Calhoun et al., 2010). Aligned with these three categories, Tedeschi and Calhoun (1996) identified five domains of potential positive changes through their development of the Post-Traumatic Growth Inventory (PTGI) measurement, including two domains on perceived changes in self (i.e., identifying new possibilities in life and perceiving personal strength), one domain on the changed sense of relationships with others (i.e., improved interpersonal relationships), and two domains on the changed philosophy of life (i.e., appreciation of life and spiritual growth). This five-domain model of PTG has received strong empirical support (Tedeschi and Calhoun, 2004; Taku et al., 2008; Konkolÿ Thege et al., 2014). This study adopted this model to examine how parents of young children perceived their changes as they tenaciously persevere through the challenging and difficult times of the pandemic. It is noteworthy that, parents reporting changes in the five domains during the pandemic does not mean that they have necessarily encountered trauma themselves. In this study, these perceived positive changes were conceptualized as general changes during a potentially traumatic socio-historical event that ubiquitously affected the large majority of the population.

Existing research that used the PTG model to examine the potentially positive changes during the COVID-19 pandemic provided preliminary support for the prevalence of these five change domains. In a qualitative cross-sectional examination of caregivers of children in Portugal and United Kingdom, Stallard et al. (2021) reported prevalent changes in improved relationships (48%), appreciation of life (22%), healthier lifestyles (22%), new possibilities (11%), and spiritual growth (15%), which were found to be related to better well-being outcomes. Using thematic analyses on qualitative, open-ended survey responses from a sample of parents in Austria and Italy, Wenter et al. (2022) found that parent’s perceived changes among their children was associated with child reduced internalizing and aggressive symptoms. These findings were coincided with conclusions from the general population facing the pandemic in Greece (Koliouli and Canellopoulos, 2021) and Spain (Vazquez et al., 2021), as well as investigations of patients and frontline nurses in China during the first COVID-19 outbreak (Cui et al., 2021; Yan et al., 2021). However, most of the existing examinations of these perceived changes during the pandemic were either descriptive, using cross-sectional data, or focusing on factors that would predict the changes. Given the unique situations of the pandemic in the U.S. and the unique challenges posed to parents of young children, an examination of the prevalence of perceived changes in this population is still needed. In addition, this study was the first, from the authors’ knowledge, that conceptualized pandemic-related changes as a factor that promoted the processes of family resilience via their promotive and protective effects on parents’ emotional well-being and children’s subsequent behavioral adjustment.

An important factor to be taken into consideration while examining pandemic-related risk and resilience processes is the long-lasting and continuously widening sociodemographic disparities based on families’ racial/ethnic and SES backgrounds (Iruka et al., 2021; Oberg et al., 2022). Households of lower incomes, as well as families of Black and Latino(a) backgrounds, were found to experience substantially more challenges across different systems, such as higher mean levels and unpredictability of material hardship (Liu et al., 2022), more barriers to accessing healthcare services, and more severe COVID-19 health consequences (Selden and Berdahl, 2020). Inequalities also existed in the health benefits of stay-at-home orders, where individuals of Black, Latino(a), or lower-income backgrounds were more likely to be essential workers, less able to follow stay-at-home regulations, and had higher risks for COVID-19 infection and hospitalization (Rogers et al., 2020). Moreover, racism and discrimination facing families of color have been found intensified during the pandemic (Chae et al., 2021). These different types and degrees of challenges facing parents of different sociodemographic backgrounds may have implications for differential change patterns among parents of different race/ethnicity or income levels. Indeed, research has well documented the diverse forms of coping and resistance that families from marginalized racial/ethnic backgrounds engage in in response to racism, including aspirational, linguistic, familial, social, navigational, and resistant capital (Yosso, 2005). Because the pandemic is a “racial macro stressor” (Seaton, 2020), it is possible that patterns of perceived changes may be particularly enhanced among marginalized groups who have demonstrated and cultivated emotional resilience and tenacity as forms of cultural capital. Indeed, limited existing PTG studies on racial/ethnic and SES differences found Black and Latino(a) individuals to report higher levels of changes than White participants, while the SES-based differences were inconsistent across studies (Barskova and Oesterreich, 2009; Yastıbaş and Karaman, 2021). However, most of these comparison studies were conducted among patients with certain medical conditions, which was a very different context in comparison to the more ubiquitously impactful COVID-19 pandemic. New comparison studies are still needed to identify racial/ethnic and SES differences in the five domains of perceived changes during such large-scale and stressful socio-historical events, in order to address the existing structural inequalities and inform targeted and tailored therapeutic and intervention practices in the face of future similar crisis events.

Overall, this study aimed to fill existing gaps in family resilience research by achieving three aims using a large national dataset of U.S. parents with young children. The first aim was to quantify the degrees to which parents of young children perceived changes during the pandemic in the five domains of new possibilities, personal strengths, improved relationships, spiritual growth, and appreciation of life. Based on previous evidence (Koliouli and Canellopoulos, 2021; Stallard et al., 2021), we hypothesized higher degrees of changes in the domains of improved relationships, moderate degrees of changes in new possibilities, personal strengths, and appreciation of life, as well as lower degrees of spiritual growth. Considering the disparities in racial/ethnic and SES backgrounds due to the prevailing inequities in the U.S. both before and during the pandemic (Iruka et al., 2021), this study also aimed to compare these differences in the five change domains. We hypothesized Black and Latino(a) parents to report more changes than non-Latino(a) White parents. No hypothesis was made regarding the degrees of changes among the other racial/ethnic parents in comparison to Black, Latino(a), and White parents due to the lack of existing evidence and the heterogeneity of this group. Because of the mixed findings on PTG differences by SES, we also refrained from making hypotheses about the differences of perceived changes by household income levels or by the intersection of race/ethnicity and income.

The second aim of this study was to examine the promotive effects of perceived changes in the five domains on families’ well-being outcomes in the overall sample. Aligned with the cascading effects proposed in the FDM (Prime et al., 2020), we specifically examined the pathways through which parents’ perceived changes affected young children’s behaviors through reducing parents’ emotional distress. Based on the FDM theoretical framing and evidence on the connections between PTG and parents’ better well-being (Stallard et al., 2021), changes in the five domains were hypothesized to indirectly reduce young children’s behavioral problems via reducing parents’ emotional distress levels. However, the lack of previous studies testing the promotive effects of specific domains on well-being outcomes prevented us from making hypotheses about the degrees of effects of these five distinctive domains.

The third aim of this study was to investigate the protective effects of perceived changes in the five domains on parents’ and young children’s emotional well-being in the overall sample. We particularly focused on the protective effects of these domains on the “chain of hardship,” where the negative impacts of material hardship mean levels and unpredictability on young children’s behavioral problems were mediated through parents’ elevated emotional distress. As perceived changes were theorized as parent functioning indicators, we hypothesized these changes to alleviate the negative impacts of material hardship mean levels and unpredictability on parents’ emotional distress in the “chain of hardship.” Similar as Aim 2, we did not make specific hypothesis about the degrees of effects of the five domains due to the lack of previous research on this topic.

## 2 Materials and methods

### 2.1 Procedures

Data of this study were drawn from the Rapid Assessment of Pandemic Impact on Development (RAPID) study, an ongoing research project launched in April 2020 that has been using brief, frequent, and online surveys to assess the pandemic impact on families of young children under 6 years old across 50 U.S. states. Participants were recruited through family-facing organization email listservs (e.g., ParentsTogether), social media advertisements (e.g., Facebook Ads), and panel services (e.g., Amazon Mechanical Turk). To be eligible, the respondent needed to be 18 years or older, a primary caregiver of at least one child under 6 years old, fluent in English or Spanish, and residing in the U.S.

RAPID surveys included the initial recruitment and ongoing follow-up assessments. Parents who were interested in participation were first asked to fill out an eligibility survey, and once determined eligible, invited to provide consent, complete the initial recruitment survey, and entered into the participant pool. Then, for each follow-up assessment, 2,000 participants in the pool were randomly selected (stratified by race/ethnicity, income levels, and geographic regions) and invited to complete the follow-up survey. Approximately 50% of invited participants were expected to respond to the invitation, resulting in about 1,000 valid responses per follow-up survey. Both initial recruitment and ongoing follow-up surveys were administered on an ongoing basis, with a weekly frequency between April and July 2020, then switched to an alternative biweekly frequency between August 2020 and December 2021, and then changed to a monthly basis since January 2022. RAPID survey contents included core and special add-in modules. Core modules, such as material hardship and emotional well-being, were key focus areas and thus included in all initial recruitment and follow-up assessments. Special add-in modules were only included in certain follow-up assessments, enabling the study with flexibility to capture numerous domains of family experiences in a timely manner during the unprecedented, fast-changing pandemic situations, while preserving short survey lengths and preventing participant fatigue. All survey responses were manually, systemically inspected to detect potential frauds, following suggestions from recently developed protocols (Ballard et al., 2019; Pozzar et al., 2020; Storozuk et al., 2020). Survey responses that were determined to be “bots” were excluded from analyses. All study procedures have been approved by the institutional review boards. Each participant received a $5 incentive after completing each survey.

### 2.2 Participants

Between April 2020 and May 2023, RAPID collected 61,129 responses from 18,583 parents with young children. For this study, we used data from a subsample of 669 parents. The selection of this subsample took two factors into consideration. First, the core focus of the current study—perceived changes in the pandemic—was assessed via PTGI as a special add-in module during April 2022. Thus, only participants who responded to this specific follow-up assessment were included in the current study. Second, the aims of this study included examining the protective effects of perceived changes on the “chain of hardship” link, where the calculation of material hardship unpredictability required at least three data timepoints obtained before/in April 2022. The subsample of 669 parents of young children met both requirements, and thus was used for further analyses.

This subsample of 669 participants included 0.60% (*n* = 4) American Indian/Alaska Native, 4.63% (*n* = 31) Asian, 8.07% (*n* = 54) Black, 9.57% (*n* = 64) Latino(a), 0.15% (*n* = 1) Native Hawaiian/Pacific Islander, 74.44% (*n* = 498) non-Latino(a) White, 1.35% (*n* = 9) multi-race, and 1.20% (*n* = 8) other racial/ethnic groups. The large majority (97.75%, *n* = 652) of responding parents were women. Based on reported household income and family size, there were 26.36% (*n* = 175) of families below 200% federal poverty level (FPL; described as lower-income in this study), 34.04% (*n* = 226) between 200% and 400% FPL (i.e., middle income), and 39.61% (*n* = 263) at or above 400% FPL (i.e., higher income). The majority (71.88%, *n* = 478) of parents had an education level at or above a Bachelor’s degree. The geographic distribution of participants was even across the four U.S. census regions: 24.89% (*n* = 166) were from the Midwest, 22.94% (*n* = 153) were from the Northeast, 25.49% (*n* = 170) were from the South, and 26.69% (*n* = 178) were from the West.

A further breakdown of race/ethnicity by income levels suggested that Black and Latino(a) families in this sample were economically more disadvantaged than households of White and other racial/ethnic backgrounds. In particular, 48.15% of Black and 41.27% of Latino(a) families, in contrast to 23.79% White and 11.76% other racial/ethnic households, had lower household income levels. In contrast, only one in four Black (25.93%) or Latino(a) (25.40%) families, versus 40.73% White and 50.98% other racial/ethnic households, fell into the higher-income category.

A comparison of demographic characteristics of the current study sample, the full RAPID sample, and corresponding national population is presented in Supplementary Table S1. Compared to the study full sample, the current sample of 669 households included fewer families of lower income (26.36% vs. 31.37%) and more families of higher income levels (39.61% vs. 32.34%). In addition, participants included in the current sample were more likely to be non-Latino(a) White (74.44% vs. 66.62%), having an education level at or above a Bachelor’s degree (71.88% vs. 54.23%), identify as woman/transgender woman (97.75% vs. 88.48%), and reside in Northeast US (22.94% vs. 16.25%) than parents in the full RAPID sample. This current sample, in comparison to the national population, also had fewer Latino(a) (9.57% vs. 17.6%) and more White (74.44% vs. 63.7%) parents, more parents with a higher-education degree (71.88% vs. 44%), and fewer households of lower income levels (26.4% vs. 34.3%). Overall, the sample used in this study was at a sociodemographically more privileged position than the RAPID full sample and the national population.

### 2.3 Measures

Given the RAPID study’s nature of brief, frequent online surveys, the number of questions on a given topic was limited to prevent participant fatigue and enhance retention. When validated measurements were available, questions that were of highest relevance to parents’ experiences during the pandemic were selected and adapted to pandemic-appropriate contexts. However, considering the unprecedented and rapidly changing conditions in the pandemic, many topics of interest did not have existing validated measures. In this case, questions were developed by the research team and then reviewed and approved by the study’s national advisory board. Overall, RAPID surveys’ selection and design of assessment items reflected efforts to optimize the balance between rigor and practicality, a core principle of this national survey study (Liu et al., 2023, September 29). For scales with multiple items, internal consistency was examined using Cronbach’s alpha in the full sample as well as subsamples by race/ethnicity and income levels (see Supplementary Table S2).

#### 2.3.1 Parents’ perceived changes in the pandemic context

Perceived changes among parents during the pandemic were assessed using an adapted version of the PTGI (Tedeschi and Calhoun, 1996) in one follow-up assessment during April 2022. PTGI was initially developed to assess post-traumatic growth and self-improvement and has been validated to evaluate personal positive changes following stressful situations (Sheikh and Marotta, 2005). In RAPID, the PTGI items were adapted to assess parents’ positive changes during the pandemic. Parents were asked to “indicate for each of the statements below the degree to which this change occurred in your life as a result of the COVID-19 pandemic” on 21 items using a six-point Likert scale, ranging from “0—I did not experience this change” to “5—I experienced this change to a very great degree.” These 21 items were classified into five factors, including personal strengths (4 items, α = 0.85), new possibilities (5 items, α = 0.80), improved relationships (7 items, α = 0.87), spiritual growth (2 items, α = 0.86), and appreciation of life (3 items, α = 0.77). Mean scores of these five domains were calculated, respectively. Example items of the personal strength domain included “I have a greater feeling of self-reliance” and “I know that I can handle difficulties.” Example items of the new possibility domain included “I have developed new interests” and “I established a new path for my life.” Example items of the improved relationship domain included “I have a greater sense of closeness with others” and “I have more compassion for others.” Example items of the spiritual growth domain included “I have a better understanding of spiritual matters” and “I have stronger religious faith.” Example quotes of the appreciation of life domain included “I changed my priorities about what is important in life” and “I have a greater appreciation for the value of my own life.” The internal consistency indicators across all five domains in the full sample were good. When examined by racial/ethnic and income-level subgroups (see Supplementary Table S2 for details), the internal consistency indicators remained at high levels, with one exception of the appreciation of life domain among the other racial/ethnic group participants being acceptable (α = 0.64).

#### 2.3.2 Parent emotional well-being

Parents’ emotional distress was assessed in all baseline and follow-up surveys as a composite score of four symptoms: depression, anxiety, stress, and loneliness. Depressive symptoms were measured using two items from the Patient Health Questionnaire-2 (Kroenke and Spitzer, 2002), including “little interest or pleasure in doing things” and “feeling down, depressed, or hopeless.” Anxiety symptoms were assessed via the Generalized Anxiety Disorder (GAD) 2-item Scale (Kroenke et al., 2007), including “feeling nervous, anxious, or on edge” and “not being able to stop or control worrying.” Responses for the depression and anxiety questions ranged from “0—Not at all” to “3—Nearly every day.” Perceived stress symptoms were captured by one item, “stress means a situation in which a person feels tense, restless, nervous, or anxious, or is unable to sleep at night because his/her mind is troubled all the time. Did you feel this kind of stress?” (Elo et al., 2003). Responses for the stress question ranged from “0—Not at all” to “4—Very much.” Lastly, loneliness was measured by one item, “I feel lonely,” from the NIH Toolbox item bank version 2.0 (Gershon et al., 2013), with responses ranging from “0—Never” to “4—Always.” The score of each of the four symptoms was first transformed to a range of 0–100, and then averaged to obtain the composite emotional distress score for each participant at each assessment. To establish the temporal precedence for hypothesis testing, each parent’s emotional distress scores obtained concurrently with the PTGI variables (i.e., April 2022) and during their first follow-up response after April 2022 (i.e., F1) were extracted for further analyses. The four emotional distress symptoms were moderately to highly correlated, 0.60 ≤ *r* ≤ 0.72, *p* < 0.001, and had good internal consistency, α_April-2022_ = 0.85, α_F1_ = 0.87. When examined by racial/ethnic and income-level subgroups, the internal consistency indicators remained good in all subgroups.

#### 2.3.3 Child emotional well-being

As RAPID’s nature of frequent brief surveys limited our capacity to include long assessment tools, we chose to include two items from the Child Behavioral Checklist (Achenbach, 2001) to assess children’s behavioral problems in all baseline and follow-up surveys. These two items, including “Too fearful and anxious” and “Fussy or defiant,” were chosen because they were broadly applicable to children of different developmental stages in the 0–5 age range and reflected tendencies of internalizing and externalizing symptoms, respectively. Parents reported these symptoms on each of the children within the age range in their household, using a response set of “0—not true,” “1—somewhat/sometimes true,” and “2—often true/very true.” The average scores across all reported children in the household were calculated to reflect children’s fear/anxiety and fussiness/defiance symptoms, respectively, at the household level. To establish the temporal precedence for further hypothesis testing, each household’s overall child fussiness/defiance and fear/anxiety symptoms obtained during their first (F1) and second (F2) follow-up responses after the April 2022 assessment were extracted for further analyses. The scores of these two symptoms were moderately correlated, *r*_F1_ = 0.40, *r*_F2_ = 0.40, *p < 0*.001.

#### 2.3.4 Material hardship experiences

Material hardship was assessed in all baseline and follow-up surveys with one item adapted from the Institute of Medicine financial strain scale (IOM, 2014): “Which of these needs have been hard to pay for in the past month? Select all that apply.” Responses included “Food,” “Housing,” “Utilities (electric, water, trash, etc.),” “Healthcare,” “Childcare,” and “Social and Emotional.” Responses included “1—Yes” and “0—No.” For each family’s each survey response, material hardship level was indicated by the number of basic needs that families had difficulty paying for (ranged 0 to 6).

As mentioned earlier in the participants section, families’ material hardship experiences assessed during and before the April 2022 survey were used in the current analyses to establish temporal precedence for hypothesis testing, and only families with at least three timepoints of data obtained at or before April 2022 were included in this study. For each family, the average material hardship level was calculated by taking the mean score of their hardship levels during the multiple responses. The material hardship unpredictability was obtained using the coefficient of variance (CV), which was the standard deviation divided by the mean of their hardship levels during the multiple responses. CV is a commonly used method to assess unpredictability (e.g., Key et al., 2017), with higher scores indicating more unpredictability.

### 2.4 Data analysis

Descriptive statistics and Pearson’s bivariate correlation coefficients of all study variables were first obtained in R (Version 4.2.0) to examine their distributions and associations. Then, to quantify the degrees to which parents perceived the five domains of changes and their sociodemographic differences (Aim 1), descriptive statistics of the five domains were obtained in the full sample, as well as subsamples by race/ethnicity, income levels, and their intersection. Independent-sample *t-*tests, ANOVA (with Tukey’s HSD) analyses were conducted in R to examine the statistical significance of racial/ethnic and income-level mean differences. For the second and third study aims, structural equation models (SEM) were constructed in M*plus* Version 8.3 using maximum likelihood estimation (Muthén and Muthén, 2017). In all SEM models mentioned below, model fit was assessed through the chi-square, the comparative fit index (CFI), and the standardized root mean residual (SRMR; Hu and Bentler, 1999).

In the dataset of 669 participants, the average missing data rate across all study variables was 10.81%. Given the study design, variables on the five change domains and material hardship had no missing data; variables assessed at F1 had 24.8% of missing data; variables assessed at F2 had substantial missing data of 54.7%. Little’s test suggested that a missing completely at random (MCAR) pattern (χ^2^[31] = 29.14, *p* = 0.56). Thus, missing data were addressed using M*plus* default full information maximum likelihood (FIML) algorithm. Although the missing data rate at F2 was high, there was evidence that FIML could yield robust, accurate estimations with a 50% missing rate when a strong auxiliary variable (i.e., a variable that correlate with the variable with high missing data at *r* = 0.50) was included (Nelson et al., 2021). The current study models included such auxiliary variables—child behavioral problems at F1—that correlated with F2 data with *r* > 0.50; thus, FIML was appropriate in this study.

To test the hypothesized promotive effects (Aim 2), an SEM model was constructed to examine the five change domains during April 2022 altogether as independent variables, parents’ emotional distress composite score during F1 as the mediator, and child fussiness/defiance and fear/anxiety symptoms at F2 as the dependent variables. The path a of this mediation model controlled for parent emotional distress assessed at April 2022, and path b of this model controlled for child corresponding symptoms assessed at F1, and all paths controlled for parents’ race/ethnicity and poverty status (i.e., below 200% FPL). The R-Mediation procedure was employed to estimate the indirect effect coefficients and confidence intervals (Tofighi and MacKinnon, 2011).

In the test of Aim 3, an SEM mediation model of the “chain of hardship” was first constructed, where the indirect effects of material hardship mean levels and unpredictability on changes in child fussiness/defiance and fear/anxiety symptoms through changes in parent emotional distress were tested. Then, five moderated mediation models were constructed to examine the moderating effects of the five change domains, respectively, on the “chain of hardship.” In particular, the moderating effects were examined using interaction analyses on path a given the assumption that perceived changes would directly buffer parents’ emotional distress. These models also controlled for parent emotional distress assessed at April 2022, child corresponding symptoms assessed at F1, and parents’ race/ethnicity and poverty status. Significant interaction effects indicated significant moderation effects, which were further probed using simple-slope tests.

## 3 Results

### 3.1 Study variables correlations

Table 1 presents the descriptive statistics and bivariate correlations of study variables. The five domains of perceived changes were strongly and positively correlated with each other (*r* ranged from 0.43 to 0.75, *p*s < 0.01). Changes in personal strengths (*r* = −0.08, *p* < 0.05) and improved relationships (*r* = −0.09, *p* < 0.05) were associated with lower levels of parent emotional distress assessed at the same timepoint. The mean level of material hardship was positively correlated with changes in new possibilities (*r* = 0.15, *p* < 0.01), personal strengths (*r* = 0.12, *p* < 0.01), spiritual growth (*r* = 0.09, *p* < 0.05), and appreciation of life (*r* = 0.11, *p* < 0.01), and positively associated with parent emotional distress (*r* ranged from 0.33 to 0.36, *p*s < 0.01) and child behavioral problems (*r* ranged from 0.15 to 0.25, *p*s < 0.01) assessed at all timepoints. The unpredictability of hardship status, although not significantly linked to any domain of changes, was positively associated with parent emotional distress at the April 2022 (*r* = 0.09, *p* < 0.05) and F1 (*r* = 0.12, *p* < 0.01) as well as child behavioral problems at F2 assessment (*r* ranged from 0.21 to 0.14, *p*s < 0.01). Higher levels of parent emotional distress were significantly associated with higher levels of child behavioral problems at all assessed timepoints (*r* ranged from 0.32 to 0.43, *p*s < 0.01).

**Table 1:** Descriptive statistics and bivariate correlations of study variables (*N* = 669).

	1	2	3	4	5	6	7	8	9	10	11	12	13	14	15	16	17
1. New possibilities (April22)	–																
2. Personal strengths (April22)	0.75^**^	–															
3. Improved relationships (April22)	0.68^**^	0.69^**^	–														
4. Spiritual growth (April22)	0.47^**^	0.46^**^	0.51^**^	–													
5. Appreciation of life (April22)	0.67^**^	0.72^**^	0.65^**^	0.43^**^	–												
6. Hardship mean level (At/Pre April22)	0.15^**^	0.12^**^	0.06	0.09^*^	0.11^**^	–											
7. Hardship unpredictability (At/Pre April22)	0.05	0.01	0.01	0.06	0.06	0.04	–										
8. Parent emotional distress (April22)	−0.02	−0.08^*^	−0.09^*^	−0.06	−0.02	0.36^**^	0.09^*^	–									
9. Parent emotional distress (F1)	0.01	−0.05	−0.08	−0.04	0.02	0.33^**^	0.12^**^	0.76^**^	–								
10. Child fussiness/defiance (F1)	−0.02	−0.01	−0.03	−0.06	0.02	0.15^**^	0.06	0.33^**^	0.39^**^	–							
11. Child fear/anxiety (F1)	0.04	0.00	0.03	0.01	0.05	0.25^**^	0.08	0.39^**^	0.43^**^	0.40^**^	–						
12. Child fussiness/defiance (F2)	−0.05	−0.05	−0.06	−0.06	0.04	0.22^**^	0.21^**^	0.32^**^	0.34^**^	0.50^**^	0.30^**^	–					
13. Child fear/anxiety (F2)	0.03	0.01	0.04	0.02	0.09	0.16^**^	0.14^**^	0.38^**^	0.37^**^	0.40^**^	0.56^**^	0.47^**^	–				
14. Black	0.12^**^	0.10^*^	0.13^**^	0.20^**^	0.14^**^	0.05	0.01	0.00	0.00	0.04	0.03	−0.04	0.00	–			
15. Latino(a)	0.14^**^	0.09^*^	0.13^**^	0.11^**^	0.12^**^	0.10^*^	0.09^*^	−0.01	0.02	0.00	0.09^*^	0.09	0.15^**^	−0.10^*^	–		
16. Other racial/ethnic groups	0.03	0.00	0.01	−0.06	0.02	0.00	−0.01	−0.06	−0.04	−0.02	0.01	−0.04	−0.04	−0.09^*^	−0.10^*^	–	
17. Poverty	0.00	−0.01	−0.09^*^	0.07	−0.04	0.40^**^	0.14^**^	0.17^**^	0.18^**^	0.08	0.13^**^	0.09	0.12^*^	0.15^**^	0.11^**^	−0.08^*^	–
Mean	1.71	2.05	1.56	1.02	2.49	0.65	0.66	32.27	34.16	0.91	0.39	0.95	0.45	0.08	0.10	0.08	0.26
*SD*	1.17	1.30	1.12	1.38	1.28	1.06	1.02	22.73	23.47	0.60	0.53	0.58	0.57	0.27	0.29	0.27	0.44

SD, Standard Deviation; April22, survey administered during April 2022, when PTGI items were assessed; F1, each participant’s first response after the April 2022 assessment; F2, each participant’s second response after the April 2022 assessment. Black, Latino(a), Other Racial/Ethnic Groups variables were all 0/1 binary variables. Poverty was also a binary variable, with “1” indicating below 200% FPL. *p < 0.05, **p < 0.01.

### 3.2 Degrees of changes and socio-demographic differences

**Figure 1:**
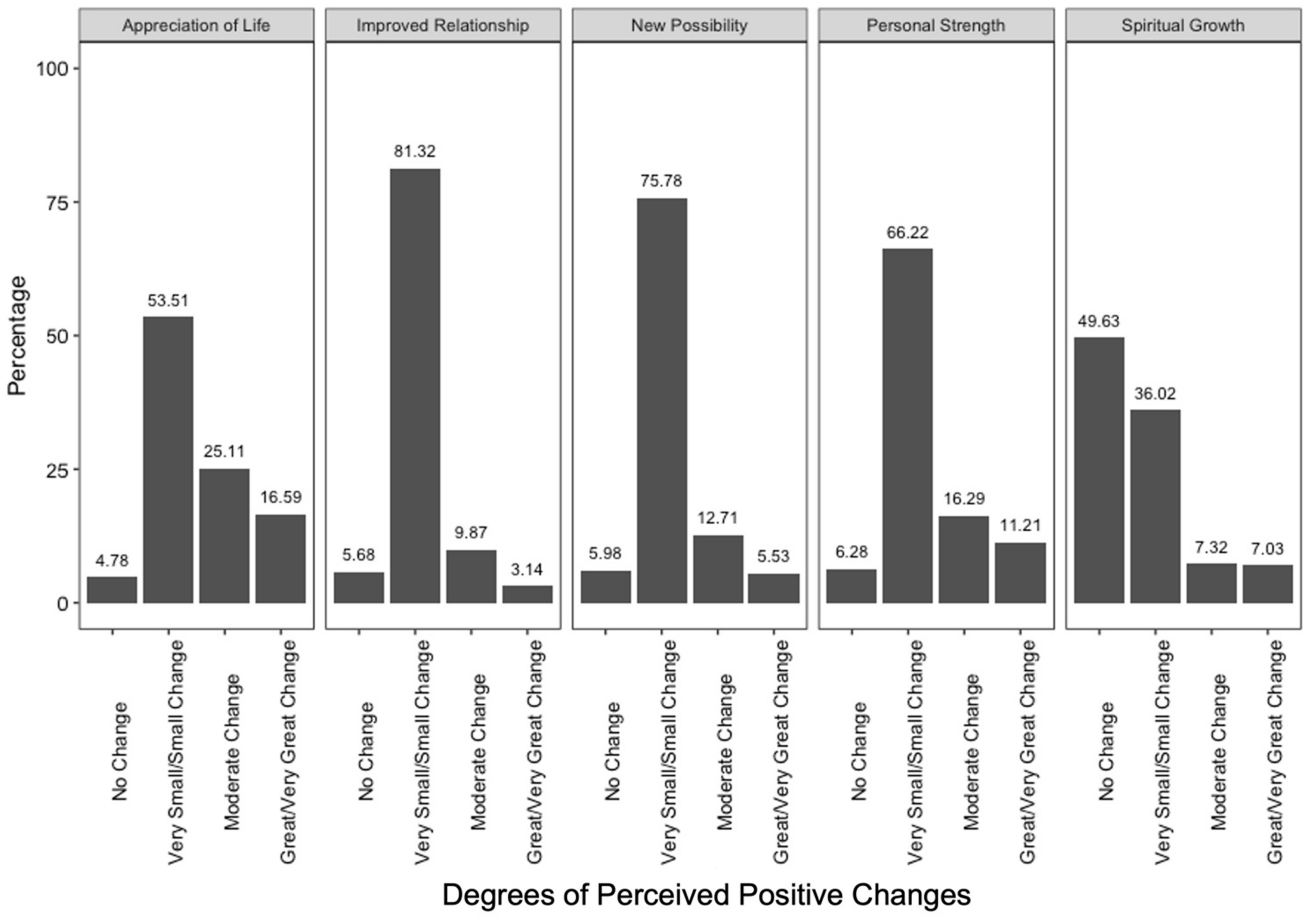
The degree to which parents of young children reported perceived changes, as presented in percentages. This figure presents the percentages of parents selecting different degrees of changes in the five domains of changes. For each domain, the mean scores ranged from 0 to 5. Mean scores of 0 were coded as “no change.” Mean scores above 0 and smaller than 3 were coded as “very small/small change,” in correspondence with the response set of “1—I experienced this change to a very small degree” and “2—I experienced this change to a small degree.” Mean scores above at/above 3 and below 4 were coded as “moderate change,” in correspondence with the response set of “3—I experienced this change to a moderate degree.” Mean scores at/above 4 were coded as “great/very great change,” in correspondence with the response set of “4—I experienced this change to a great degree” and “5—I experienced this change to a very great degree”.

Figure 1 presents the percentages of parents reporting different degrees of changes in the five domains of changes. The percentages of parents reporting moderate-to-large degrees of changes were the highest for the appreciation of life domain (41.70%), followed by personal strengths (27.50%), new possibilities (18.24%), spiritual growth (14.35%), and improved relationships (12.98%). Overall, 49.93% of parents reported moderate-to-large degrees of changes in at least one domain.

**Figure 2:**
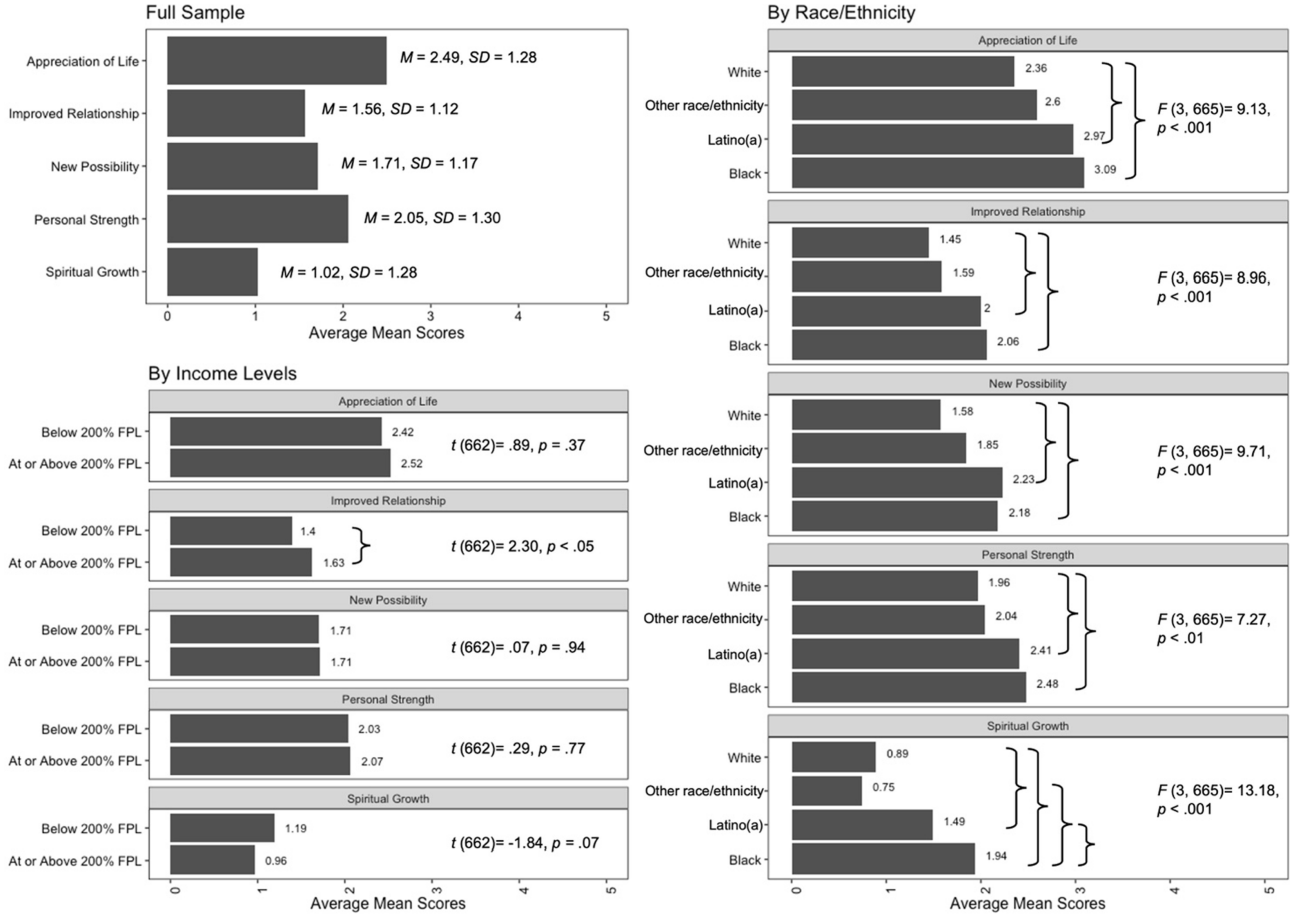
Mean scores of perceived change domains among the full sample and comparisons by of race/ethnicity and income levels, respectively. In each panel, the mean scores of each domain for the full sample or among each sociodemographic (racial/ethnic and income levels) group are presented. In the panel that shows the comparison by income levels, results of independent-sample *t*-tests are presented. In the panel that shows the comparison by race/ethnicity, results of ANOVA are presented. For the two comparison panels, the curly brackets } indicate group comparisons that showed statistically significant differences. For the mean differences, see descriptions in the Results section.

Figure 2 shows the mean levels of changes in the five domains and comparisons by race/ethnicity and income levels, respectively. Comparisons by income indicated largely comparable change levels between households of lower and middle-to-higher income levels in appreciation of life, new possibilities, personal strengths, and spiritual growth. However, parents of middle-to-higher income levels reported significantly more improved relationships than parents of lower income levels, 95% CI*_Mdifference_*[0.03, 0.52], *p* < 0.05. Mean score comparisons by race/ethnicity revealed significant differences mainly between Black-White and Latino(a)-White parents. Compared to White parents, Black parents reported significantly higher levels of all five domains: appreciation of life, 95% CI*_Mdifference_*[0.26, 1.20], *p* < 0.001; improved relationships, 95% CI*_Mdifference_*[0.21, 1.02], *p* < 0.001; new possibilities, 95% CI*_Mdifference_*[0.18, 1.03], *p* < 0.01; personal strengths, 95% CI*_Mdifference_*[0.03, 0.99], *p* < 0.05; and spiritual growth, 95% CI*_Mdifference_*[0.55, 1.54], *p* < 0.001. Similarly, Latino(a) parents, in comparison with White parents, also exhibited significantly higher levels of all five domains: appreciation of life: 95% CI*_Mdifference_*[0.19, 1.05], *p* < 0.01; improved relationships: 95% CI*_Mdifference_*[0.18, 0.93], *p* < 0.001; new possibilities: 95% CI*_Mdifference_*[0.25, 1.04], *p* < 0.001; personal strengths: 95% CI*_Mdifference_*[0.003, 0.89], *p* < 0.05; and spiritual growth: 95% CI*_Mdifference_*[0.14, 1.06], *p* < 0.01. Moreover, Black (95% CI*_Mdifference_*[0.52, 1.86], *p* < 0.001) and Latino(a) (95% CI*_Mdifference_*[0.14, 1.06], *p* < 0.01) parents reported higher levels of spiritual growth than families of the other racial/ethnic backgrounds.

**Figure 3:**
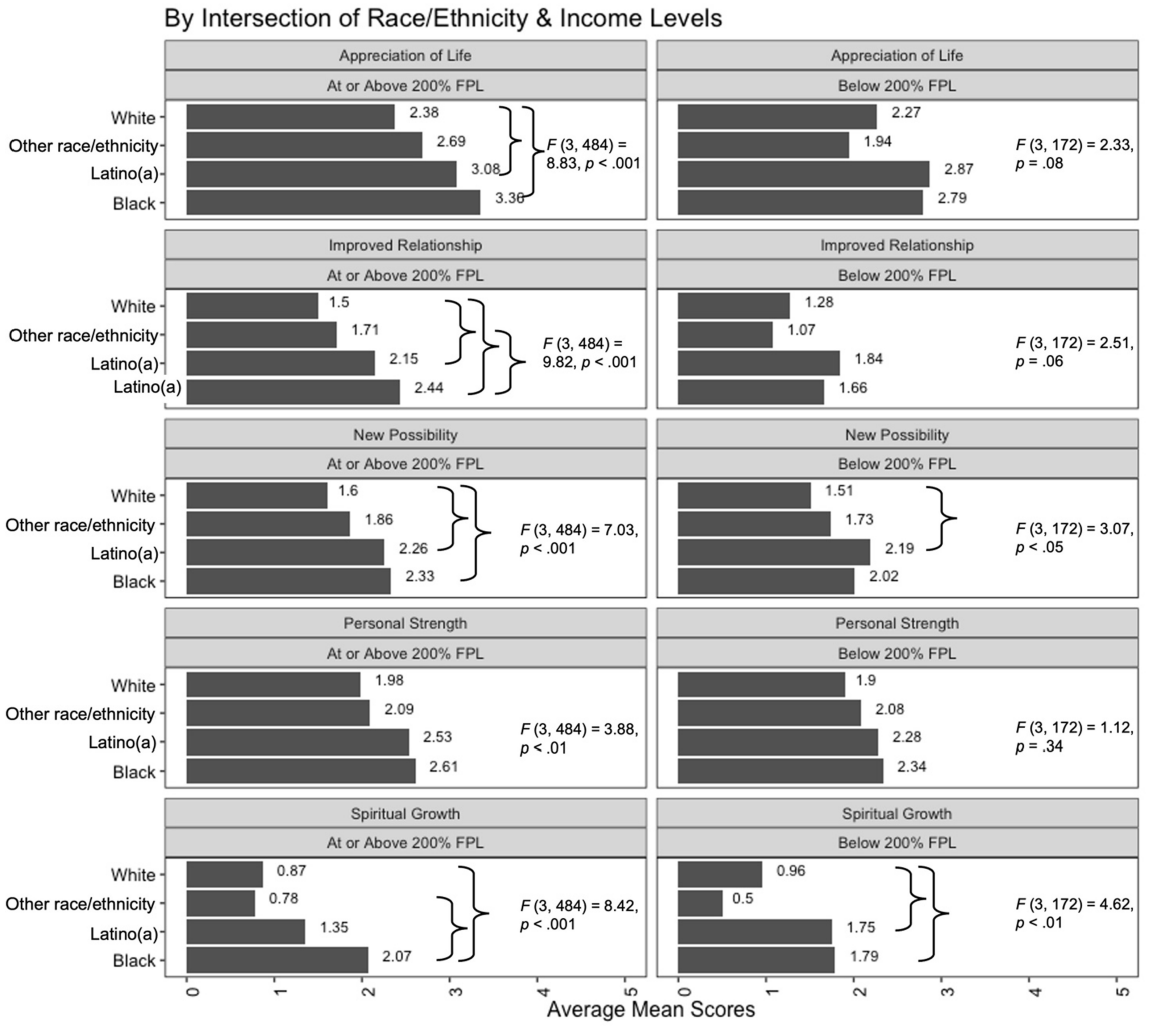
Comparisons of mean scores of perceived change domains by the intersection between race/ethnicity and income levels. In each panel, results of ANOVA that examined racial/ethnic differences within the income-level category (lower or middle-to-higher) are presented. The curly brackets} indicate group comparisons that showed statistically significant differences. For the mean differences, see descriptions in the Results section.

We further examined the mean level differences of perceived changes by the intersection of race/ethnicity and income levels (Figure 3). Comparisons by income levels within each race/ethnicity group suggested largely comparable degrees of changes between lower- and middle-to-higher-income households in the five domains, regardless of the parents’ racial/ethnic backgrounds. The exception was that among Black (95% CI*_Mdifference_*[0.03, 1.52], *p* < 0.05) and White (95% CI*_Mdifference_*[0.01, 0.44], *p* < 0.05) families, higher-income parents reported more improved relationships than lower-income ones.

For comparisons by race/ethnicity within each income-level group, among families of middle-to-higher income levels, Black parents reported significantly more changes than White parents in four domains: appreciation of life, 95% CI*_Mdifference_*[0.36, 1.60], *p* < 0.001; improved relationships, 95% CI*_Mdifference_*[0.39, 1.48], *p* < 0.001; new possibilities, 95% CI*_Mdifference_*[0.16, 1.31], *p* < 0.01; and spiritual growth, 95% CI*_Mdifference_*[0.53, 1.88], *p* < 0.001. Black parents of middle-to-higher income also reported more changes in personal strengths than White parents of the same income levels, but this difference was only approaching statistical significance, 95% CI*_Mdifference_*[−0.02, 1.27], *p* = 0.06. Meanwhile, middle-to-higher-income Latino(a) parents, in comparison with White parents, reported significantly more changes in appreciation of life (95% CI*_Mdifference_*[0.16, 1.25], *p* < 0.01), improved relationships (95% CI*_Mdifference_*[0.16, 1.13], *p* < 0.01), and new possibilities (95% CI*_Mdifference_*[0.15, 1.16], *p* < 0.01), as well as more changes in personal strength that was approaching significance (95% CI*_Mdifference_*[−0.02, 1.12], *p* = 0.06). In addition, middle-to-higher-income Black parents reported significantly more changes in improved relationships (95% CI*_Mdifference_*[0.06, 1.40], *p* < 0.05) and spiritual growth (95% CI*_Mdifference_*[0.47, 2.12], *p* < 0.001) than parents of the other racial/ethnic groups with the same income levels.

Among households of lower income levels, similar difference patterns were discovered where Black and Latino(a) parents reported more changes in the five domains than parent of White and other racial/ethnic backgrounds. In particular, Black parents of lower incomes indicated significantly more spiritual growth than White parents of lower incomes, 95% CI*_Mdifference_*[0.05, 1.61], *p* < 0.05. Lower-income Latino(a) parents, compared to White parents, also reported significantly more changes in new possibilities (95% CI*_Mdifference_*[0.01, 1.37], *p* < 0.05) and spiritual growth (95% CI*_Mdifference_*[0.01, 1.57], *p* < 0.05). However, the other mean level differences were not statistically significant, likely due to the smaller subsample sizes and smaller degrees of differences.

### 3.3 Promotive effects of perceived changes on parent and child well-being

**Figure 4:**
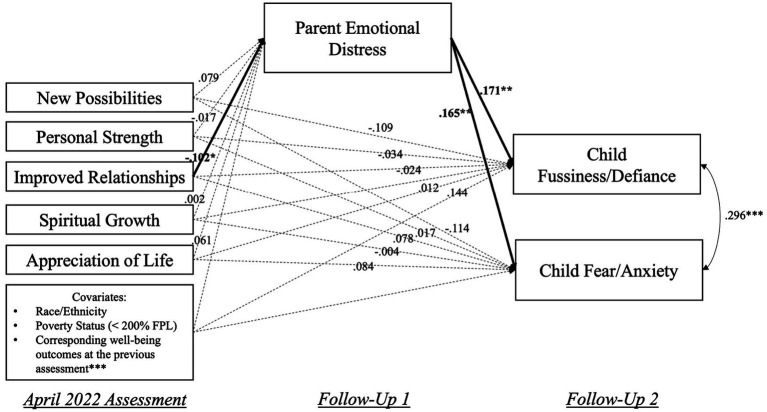
Visualization of the SEM model that examined the promotive indirect effects of perceived change domains on decreases in child behavioral problems through reducing parent emotional distress. April 2022 assessment was when PTGI items were assessed; Follow-Up 1 = each participant’s first response after the April 2022 assessment; Follow-Up 2 = each participant’s second response after the April 2022 assessment. For covariates, race/ethnicity included three binary (0/1) variables indicating participants’ being Black, Latino(a), or other non-White racial/ethnic groups; Poverty was also a binary variable, with “1” indicating below 200% FPL. Solid lines indicate statistically significant associations, and dotted lines indicated non-significant associations. *p < 0.05, **p < 0.01, ***p < 0.001.

The SEM results on the promotive indirect effects of the five change domains on child behavioral problems through parent emotional distress are presented in Figure 4 (see Supplementary Table S3 for model coefficients). This model had acceptable fit indices: χ^2^(36) = 97.00 (*p* < 0.001), CFI = 0.917, SRMR = 0.041. Perceived changes in improved relationships during the pandemic were significantly associated with decreases in parent emotional distress, β = −0.102, *p* < 0.05, which were further linked to decreases in child fussiness/defiance (β = 0.171, *p* < 0.01) and fear/anxiety (β = 0.165, *p* < 0.01) symptoms. The indirect effects of improved relationships on decreases in child fussiness/defiance (α*β = 0.017, 95% CI [−0.018, −0.001], *p* < 0.05) and fear/anxiety (α*β = 0.017, 95% CI [−0.018, −0.001], *p* < 0.05) symptoms through decreases in parent emotional distress were found statistically significant. However, the other four change domains were found not significantly associated with changes in parent emotional distress or child behavioral problems.

### 3.4 Protective effects of perceived changes on the “chain of hardship”

#### 3.4.1 Establishing the “chain of hardship”

**Figure 5:**
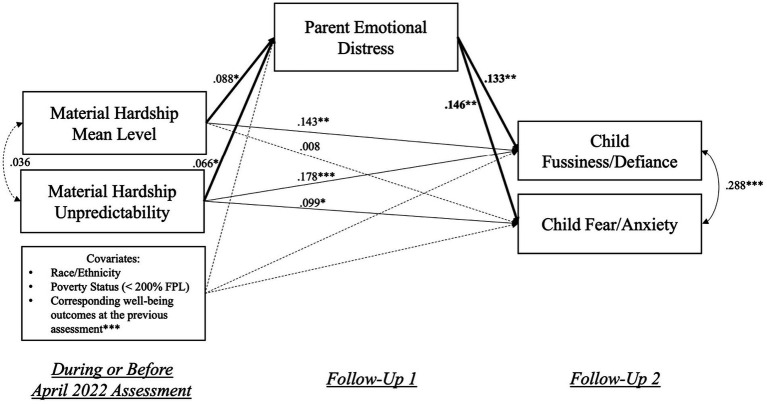
Visualization of the SEM model examining the “Chain of Hardship” hypothesis. April 2022 assessment was when PTGI items were assessed; Material hardship information obtained during or before the April 2022 assessment was aggregated and used in analyses; Follow-Up 1 = each participant’s first response after the April 2022 assessment; Follow-Up 2 = each participant’s second response after the April 2022 assessment. For covariates, race/ethnicity included three binary (0/1) variables indicating participants’ being Black, Latino(a), or other non-White racial/ethnic groups; Poverty was also a binary variable, with “1” indicating below 200% FPL. Solid lines indicate statistically significant associations, and dotted lines indicated non-significant associations. *p < 0.05, **p < 0.01, ***p < 0.001.

Figure 5 present the SEM “chain of hardship” model, where the hypothesis on the indirect effects of material hardship mean levels and unpredictability on increases in child behavioral symptoms through elevated parental emotional distress were tested (see Supplementary Table S4 for model coefficients). This model had good fit indices: χ^2^(36) = 44.15 (*p* < 0.001), CFI = 0.955, SRMR = 0.029. The results suggested that higher material hardship mean levels (β = 0.088, *p* < 0.05) and unpredictability (β = 0.066, *p* < 0.05) were both linked to increases in parent emotional distress, which were further associated with increases in child fussiness/defiance (β = 0.133, *p* < 0.05) and fear/anxiety (β = 0.146, *p* < 0.01) symptoms over time. The indirect effects of hardship mean levels (α*β = 0.012, 95% CI [0.001, 0.013], *p* < 0.05) and unpredictability (α*β = 0.009, 95% CI [0.0003, 0.011], *p* < 0.05) on child fussiness/defiance through parent emotional distress were both statistically significant. The indirect effects of hardship mean levels (α*β = 0.013, 95% CI [0.001, 0.016], *p* < 0.05) and unpredictability (α*β = 0.010, 95% CI [0.001, 0.013], *p* < 0.05) on child fear/anxiety through parent emotional distress were also statistically significant. These findings supported the “chain of hardship” hypothesis.

#### 3.4.2 Protective effects of parents’ perceived changes

**Figure 6:**
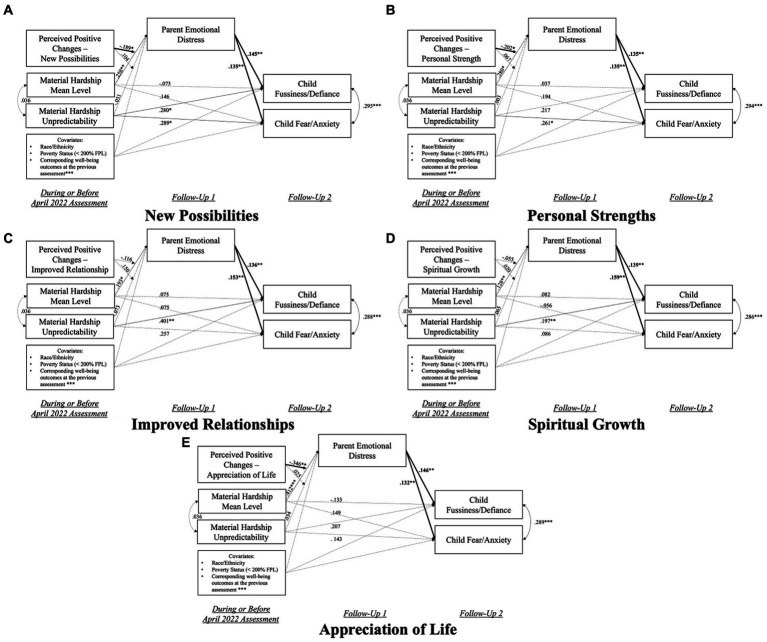
Visualization of the SEM models that examined the protective effects of perceived change domains on the “Chain of Hardship”. **(A)** The moderating effects of changes in the new possibilities domain. **(B)** The moderating effects of changes in the personal strengths domain. **(C)** The moderating effects of changes in the improved relationships domain. **(D)** The moderating effects of changes in the spiritual growth domain. **(E)** The moderating effects of changes in the appreciation of life domain. April 2022 assessment was when PTGI items were assessed; Follow-Up 1 = each participant’s first response after the April 2022 assessment; Follow-Up 2 = each participant’s second response after the April 2022 assessment. For covariates, race/ethnicity included three binary (0/1) variables indicating participants’ being Black, Latino (a), or other non-White racial/ethnic groups; Poverty was also a binary variable, with “1” indicating below 200% FPL. Solid lines indicate statistically significant associations, and dotted lines indicated non-significant associations. *p < 0.05, **p < 0.01, ***p < 0.001.

Figure 6 presents the results of the five models that examined the protective effects of the five change domains on the “chain of hardship” associations (see Supplementary Table S5 for model coefficients). All models had good fit indices: χ^2^(36) ranged from 45.302 to 48.795 (*p* < 0.001), CFI ranged from 0.953 to 0.957, SRMR ranged from 0.023 to 0.024. The findings suggested that perceived changes among parents of young children in new possibilities (β = −0.189, *p* < 0.05), personal strengths (β = −0.202, *p* < 0.05), and appreciation of life (β = −0.346, *p* < 0.01) significantly buffered the impact of material hardship mean levels on parents’ elevated emotional distress. However, this link was not significantly moderated by changes in improved relationships (β = −0.116, *p* = 0.144) or spiritual growth (β = −0.055, *p* = 0.263). No significant moderation effect of the perceived change domains on the associations between material hardship unpredictability and parents’ elevated emotional distress was discovered either. Moreover, higher levels of parent emotional distress remained to be significantly linked to increases in child fussiness/defiance and fear/anxiety symptoms (*p* < 0.01) in all five models.

**Figure 7:**
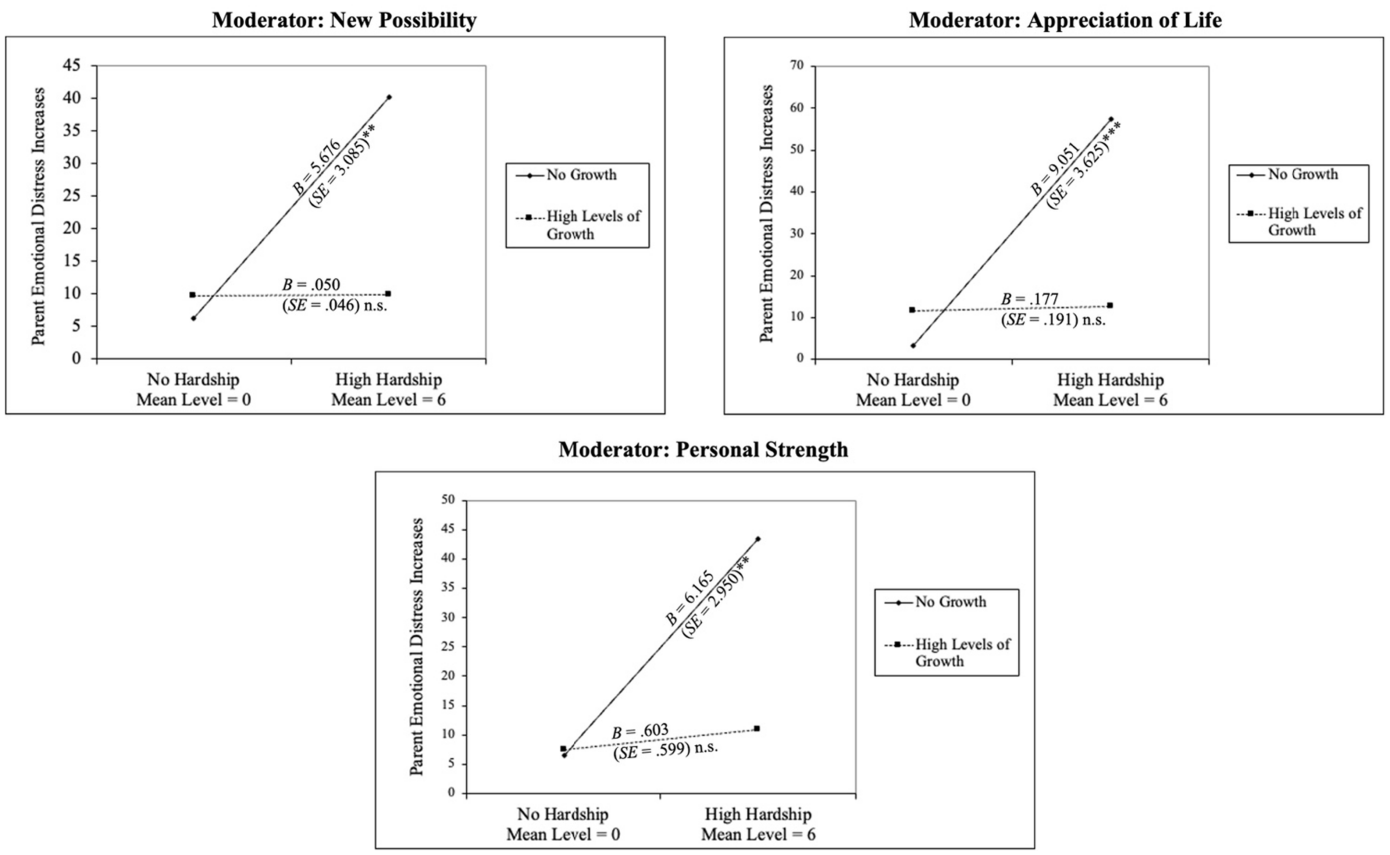
Simple slope probe on the significant moderating effects of perceived change domains on the associations between material hardship mean levels and parents’ elevated emotional distress. In each figure, the associations between material hardship levels and changes in parents’ emotional distress were plotted at two levels of the moderator (i.e., Perceived Changes)—no change (moderator = 0—“I did not experience this change”) and high level of changes (moderator = 4—“I experienced this change to a great degree”). The full range of the independent variable—material hardship mean levels—were probed. Simple slope unstandardized coefficients *B* and standard errors *SE,* along with their significance levels, are presented in each figure. **p < 0.01, *** *p* < 0.001, *n.s.* = non-significant (*p* > 0.05).

Figure 7 presents the interpretation of the three significant moderation effects. In each probed figure, the associations between the material hardship mean level and changes in parents’ emotional distress were plotted at two levels of the moderator (i.e., parents’ perceived positive changes)—no change (moderator level at 0—“I did not experience this change”) and high level of changes (moderator level at 4—“I experienced this change to a great degree”). These interpretation figures presented similar interaction patterns of perceived changes and material hardship mean levels in relation to increases in parental emotional distress. For parents who reported no change in the three domains, higher mean levels of material hardship were significantly associated with increases in parent emotional distress. In contrast, for parents who reported high degrees of changes in the three domains, the links between material hardship mean levels and increases in parent emotional distress were not statistically significant anymore. In other words, parents’ perceived changes in the domains of new possibilities, personal strengths, and appreciation of life significantly buffered the negative impact of material hardship mean levels on their emotional distress. These buffering effects were found among parents who reported mean scores of new possibilities at or above 2.23 (30.2% of parents), mean scores of personal strengths at or above 2.74 (32.7% of parents), and mean scores of the appreciation of life domain at or above 2.89 (41.7% of parents). Thus, parents’ perceived changes in new possibilities, personal strengths, and appreciation of life to a moderate degree or above (corresponding to the mean scores at/above 3) could sufficiently mitigate the negative impact of material hardship mean levels on emotional well-being among parents of young children.

## 4 Discussion

The purpose of the current study was to examine family resilience processes during the COVID-19 pandemic through the lens of parents’ perceived positive changes, previously identified in the PTG model (Tedeschi and Calhoun, 2004; Calhoun et al., 2010; Tedeschi et al., 2018). This study examined the extent to which U.S. parents with young children perceived positive emotional and mental changes in themselves during the pandemic, the sociodemographic differences in these areas of changes, and the roles (i.e., as promotive and protective factors) of these change domains in the family resilience process. We first found that half of the parents reported moderate-to-large degrees of changes, and the most prevalent changes were observed in the domain of appreciation of life, followed by personal strengths, new possibilities, improved relationships, and lastly, spiritual growth. Black and Latino(a) parents, in particular, reported more changes in all five domains than White parents, and more spiritual growth than parents of the other race/ethnicity. This racial/ethnic difference pattern largely retained among families of lower- and middle-to-higher income levels, respectively. Second, our findings suggested that parents’ perceived changes in improved relationships had significant promotive effects on families’ emotional well-being, where this domain of change indirectly reduced young children’s fussiness/defiance and fear/anxiety symptoms, on a household level, through reducing parents’ emotional distress. Lastly, parents’ perceived changes in personal strengths, new possibilities, and appreciation of life significantly buffered the “chain of hardship,” particularly the impact of material hardship mean levels (but not unpredictability) on parents’ emotional distress. For parents who perceived moderate-to-large degrees of changes in these domains, material hardship mean levels were no longer significantly linked to increases in emotional distress, which further protected children from fussiness/defiance and fear/anxiety symptoms.

### 4.1 Interpretation of study findings

In the current investigation, half of the parents reported moderate-to-large degrees of changes across the five domains, and the prevalence of the five domains ranged between 13 and 42%. The overall prevalence of perceived changes in the pandemic, which was largely consistent with meta-analyses findings on PTG prevalence (53%) among individuals who experienced traumatic events (Wu et al., 2019), highlighted the tenacity and perseverance that parents, as well as the family systems, exhibited during the COVID-19 pandemic, signaling family resilience. Despite the numerous challenges such as financial hardships, child care disruptions, lack of healthcare access, and social isolation, considerable percentages of parents reported positive changes in their attitudes, values, and interpersonal relationships. However, inconsistent with our hypothesis established on existing research (Koliouli and Canellopoulos, 2021; Stallard et al., 2021), we found the highest degree of changes in the appreciation of life, rather than the improved relationships domain. This difference could be due to survey timing and contextual differences. The two previous studies were both conducted during the initial lockdown (before May/June 2020) in European countries when the duration of pandemic disruptions was still fairly brief compared to the current study sample (assessed 2 years into the pandemic). The prolonged exposure to pandemic disruptions—especially social isolation—in this sample, as well as other concurrent events in the U.S. (e.g., intensified racism, political polarization), could reduce the chance for improved interpersonal relationships. Meanwhile, being 2 years into the pandemic, parents in the current sample might have had more time to reflect on their life priorities and philosophies, and thus reported higher degrees of changes in the appreciation of life domain.

Black and Latino(a) parents were found to exhibit more changes during the pandemic than White parents across all five domains and more spiritual growth than parents of the other racial/ethnic backgrounds, regardless of household income levels. These racial/ethnic differences were consistent with existing evidence that documented more PTG among Black and Latino(a) individuals in various populations after traumatic experiences (Barskova and Oesterreich, 2009; Yastıbaş and Karaman, 2021). The more prevalent changes among Black and Latino(a) parents of young children may have stemmed from their tighter bonds with their communities and extended families (Mulvaney-Day et al., 2007; Taylor et al., 2013). Such forms of social support and kinship bonds are critical in the face of ongoing stressors due to racism (Stamps et al., 2021), and both familial and social capital have been recognized as accumulated assets and resources that families of color have cultivated to cope from and resist racism and systemic exclusion (Yosso, 2005). Indeed, in the conceptual model of PTG, Tedeschi and Calhoun (2004) emphasized the key roles social support played in enhancing individuals’ tolerance of distress, sustaining cognitive processing, and promoting positive changes. It is possible that for Black and Latino(a) parents in this sample, strong family and community connectedness provided essential social network and support for families to cope with the pandemic challenges, cognitively process these experiences, find the “silver lining” in difficult situations, and perceive positive changes despite challenging circumstances. Another possible explanation is related to Black and Latino(a) parents’ ongoing and pernicious exposure to systemic racism, discrimination, and race/ethnicity-based trauma. Persistent, recurrent, and cumulative exposures of racism and discrimination constitute traumatic experiences (Chin et al., 2020; Saleem et al., 2020), and Black and Latino(a) parents have long had to persevere through these traumatic experiences and continue supporting their families and young children. In this tenacious process of resistance, Black and Latino(a) parents might have been able to develop the processes of perceiving positive changes through deliberative rumination, creating redemptive narratives, and discovering deeper meaning prior to the pandemic (Chin et al., 2023). Since racial/ethnic discrimination and inequalities were key components of challenges facing parents of color during the pandemic, these parents might have been able to leverage their previous experiences to better cope with the pandemic-posed stressors and perceive more positive changes, even though they were disproportionally, negative affected by the pandemic challenges.

Parents’ changes in the domain of improved relationships were found to be directly associated with lower emotional distress among themselves, which fully mediated the effects of these changes on their children’s fussiness/defiance and fear/anxiety symptoms, suggesting a promotive effect of improved relationships on family well-being. This finding was aligned with the FDM (Prime et al., 2020), which underscored the roles of family interactions and dynamics in affecting parents’ emotional well-being and having cascading impacts on children’s adjustment in the context of disruptions infiltrated by the pandemic. Given the ubiquity of pandemic disruptions in the U.S., individuals in a family system often share the same difficulties and challenges. These shared struggles could increase a parent’s sense of compassion towards their spouse, children, and other family members, and facilitate the establishment of tighter family connections (Tedeschi and Calhoun, 2004). Stronger family connectedness further encompasses trust, understanding, and support among family members, creating a support network for parents where they have sufficient resources to cope with stressors and reduce emotional distress. Such family context also provides young children with a nurturing environment that meets emotional needs, and is found robustly associated with healthy child development (Chu et al., 2010).

We also found that parents’ perceived changes in new possibilities, personal strengths, and appreciation of life could alleviate the negative “chain of hardship” and protect parents and young children from worsened emotional and behavioral well-being. In particular, some parents might learn about new supportive resources and services they could rely on (Stephens et al., 2021), find new activities for the family in the lockdown (Hazlehurst et al., 2022), or discover new career or educational paths (Akkermans et al., 2020). They might also be compelled to handle challenging situations that they had not encountered before, and through this process, discover their strengths and capabilities to successfully navigate through difficulties. In these perceived changes of parents themselves, identifying new possibilities could present parents a brighter outlook towards the future, and perceiving stronger personal strengths might equip them with confidence and self-efficacy to handle future challenges. Both aspects of changes could allow parents of young children to maintain healthy emotional well-being, despite the current struggles with material hardships. Moreover, witnessing the widespread COVID-19 infections, hospitalizations, and deaths in-person or via media may prompt parents to reconsider and radically change the priorities in their lives, increase their appreciation for what they still have in life, and recognize the value of things they used to take granted for (Asmundson et al., 2021). These changes in parents’ philosophy of life, specifically, increased gratitude, have been suggested as an essential factor for sustaining emotional well-being during the difficult circumstances of the pandemic (Jans-Beken, 2021). Indeed, a randomized controlled trial has identified increasing the appreciation of life through gratitude exercises as an effective intervention strategy to enhance adults’ mental well-being and reduce their depression, anxiety, and stress symptoms (Kloos et al., 2022).

### 4.2 Further discussions on PTG and resilience in the pandemic context

Without assessing trauma in this study, parents reporting perceived changes aligned with the PTG model did not imply that they were necessarily exposed to trauma themselves during the pandemic. Rather, these changes were assessed as parents’ general positive functioning during a potentially traumatic large-scale event that ubiquitously impacted most of the population. Our own data showed that, between April 2020 and April 2022, 98.5% of parents in this study reported experiences of at least one out of nine stressors in aspects of COVID-19 health consequences (41.9% infected; 1% hospitalized; 76.9% knowing someone hospitalized/died), economic hardships (53.1% reporting material hardship; 38.3% experiencing reduced employment), child care challenges (48.6% having difficulty finding care; 38.3% experiencing disruptions), and healthcare challenges (81.5% parents delaying own care, 39.2% delaying child preventive care). The average number of stressors was 4.5 (*SD* = 1.8). These data provided further context on the extremely high prevalence of pandemic stressors, which essentially constituted a collective traumatic event for families of young children (Holman et al., 2023). In this context, we could ascertain PTG as an appropriate framework to conceptualize positive changes during the pandemic, even though trauma was not directly assessed in the RAPID dataset.

As illustrated in Tedeschi and Calhoun (2004), the process of developing positive changes involves cognitive engagement and processing. After exposure to potentially traumatic events that challenges the individual’s understanding of the world, the individual first cognitively engages in making sense of the event itself and its meaning in their own life, then adopts cognitive processing (e.g., reappraisal, narrative development) to reconstruct these meanings, and then achieves possible changes (Henson et al., 2021). Factors such as *meaning making* and *optimism*, which have been widely reported in literature to play a role in resilience, may contribute to these positive changes as indirect functions of cognitive engagement and processing. In particular, meaning making—the process of individuals’ interpreting stressful situations and seeking restoration of their meanings—has been found as a critical process in individuals’ adaptation to stressful life events (Park, 2010), and in the PTG model (Tedeschi and Calhoun, 2004), directly related to the cognitive engagement process that entails reappraising the experience, grieving losses, and accepting changes (Zeligman et al., 2018). Additionally, optimism—a personality trait of the tendency to have positive expectancies for the future (Carver et al., 2010)—may contribute to these perceived changes via its functions on the cognitive processing of stressful events (Henson et al., 2021). Optimism has been found moderately correlated with positive changes from traumatic experiences (Helgeson et al., 2006; Prati and Pietrantoni, 2009), and is considered to exert its effect on these changes through enhancing the individual’s capacities to manage the demands of a potentially traumatic event (Benight and Bandura, 2004). Optimists were found better able to adopt adaptive coping strategies (e.g., positive reappraisal and seeking support) in stressful and potentially traumatic situations (Zoellner and Maercker, 2006), and this adaptive coping has been found as a mechanism underlying the development of positive changes from difficult, traumatic circumstances (Mattson et al., 2018). Based on Tedeschi and Calhoun (2004), optimism may allow individuals to better disengage from unachievable goals and concentrate on the most important things in a traumatic experience, which is critical for the cognitive processes that render perceived positive changes.

The process of individual development of positive changes from challenging situations might be aligned with the *stress inoculation* processes posited to underlie resilience, which suggest that exposure to moderately stressful yet controllable situations could prepare individuals to better respond to future stressful events (Ashokan et al., 2016). Similarly, in a model of psychological preparedness (Janoff-Bulman, 2004), post-traumatic positive changes is posited to help trauma survivors less traumatized by future challenging events. Meta-analytic evidence on the associations between post-traumatic positive changes and PTSD suggests a curvilinear correlation, where increases in PTSD symptoms are initially associated with increases in positive changes up to a threshold, and then the association becomes negative (Shakespeare-Finch and Lurie-Beck, 2014). As such, the common tenet of stress inoculation and the psychological preparedness models lies in that exposure to moderate levels of stress may help individuals better prepare for future stressors, and thus are related to positive outcomes.

Another important factor to consider in the developmental course of perceived positive changes during the pandemic is timing. Existing longitudinal studies seem to suggest an overall stability of these post-traumatic positive changes over time when accompanied with concurrent stressors or PTSD (Dekel et al., 2012; Tsai et al., 2016). In this study, parents of young children had been exposed to 2 years of pandemic stressors at the assessment of their perceived changes. The prolonged, continuous disruption of the pandemic exposed parents to continuous stressors; to cope with these stressors, parents might have to attain and/or sustain positive changes in their perceptions and attitudes, so that they could continue supporting their families. Further, examination of the longitudinal courses of these perceived changes reveals differential trajectories of different aspects: Within 2 years after trauma, changes in the form of a merely positive illusion (and thus not promoting better mental health) are expected to decrease over time, while constructive changes implicated in mental health symptom reduction are hypothesized to continue growing over time (Zoellner and Maercker, 2006; Liu et al., 2017). This two-year period coincides with the current study’s timeframe. As such, we anticipate the perceived changes discovered in this study among parents of young children to reflect constructive changes sustained via continuous pandemic stressors.

In summary, the five domains of perceived changes from the PTG model are useful lenses to help researchers understand the processes underlying family resilience. The PTG model and resilience theories share common conceptual propositions, including the acknowledgement that not all adverse, potentially traumatic experiences inevitably lead to negative outcomes and the focus on positive adaptations following exposures to these stressful experiences. As discussed above, many resilience-related constructs (e.g., meaning making, optimism) and processes (e.g., stress inoculation) also play a role in individual development of PTG. Meanwhile, there are clear distinctions between PTG and resilience models. While PTG specifically focuses on *individual* perceptions and indicates individual functioning, resilience highlights the *multi-system* nature of positive adaptations and emphasizes the *processes* of systemic adaptation through the interactions of its multiple components. Perceiving positive changes indicated in the PTG model is not equivalent to demonstrating resilience, as some of these changes (especially illusionary ones) might be transient, and only constructive changes could translate into better mental health outcomes. With these conceptual similarities and distinctions, perceived changes consistent with the PTG model may represent individual functioning that could facilitate systemic processes of resilience following exposure to stressful events.

### 4.3 Implications

Even though the COVID-19 pandemic has been declared over, many families of young children are still struggling with its residual consequences, such as continued child care crisis, financial hardships, and race/ethnicity- and SES-based inequalities. Therapeutic and intervention efforts aimed to address these challenges could still incorporate concepts related to these perceived positive changes (e.g., meaning making, optimism) to facilitate parents’ development or maintenance constructive changes. Beyond these efforts, not all parents perceived positive changes during the pandemic, and policies and programs targeting families’ economic hardships and instabilities are still urgently needed. Programs could consider distribute assistance in a frequent, regular manner (e.g., monthly Child Tax Credits payments), and incorporate contents that help parents cope with income volatility, in order to help families to not only improve their overall financial situation, but also attain more stability over time. In policymaking, address disparities in economic hardships and instability based on race/ethnicity and SES through increasing equal employment opportunities, protecting employees’ job security, and expanding unemployment benefits is still a high priority.

Although this study was conducted in the context of the COVID-19 pandemic, its findings are applicable to other socio-historical events that share similar characteristics, such as those that are highly stressful, unpredictable, and eliciting multi-system disruptions (e.g., natural disasters, geopolitical conflicts). In particular, this study revealed that positive changes perceived by parents of young children in such large-scale, disruptive socio-historical events could serve as promotive and protective factors to cultivate family resilience and enhance emotional well-being of both parents themselves and their young children. In the face of future similar events, therapeutic and intervention practices could incorporate these change domains to help families cope with concurrent stressors. These practices may include helping parents identify emerging new opportunities in life, enhancing parents’ self-efficacy in handling challenging situations, guiding parents to practice gratitude and reprioritize important things, and finding chances to spend family time together and enhance family connectedness.

It is important to note that, even though this study highlighted the resilience of a family system in a large-scale stressful event, this resilience does not negate the negative impacts of severe hardships experienced by households of young children, especially families of color and ones with lower income levels. For instance, it is clear to us that Black families faced more financial hardships (Ibekwe-Okafor et al., 2023) and greater mortality (Golestaneh et al., 2020) during the pandemic. Therefore, we could not expect parents’ reported positive emotional changes as framed using the PTG model to have necessarily reduced the distress induced by the week-to-week and month-to-month uncertainties in the family financial situations. As financial uncertainties and instabilities remain to be a challenge for families with young children, their negative effects on parents’ and young children’s emotional well-being remain to be concerning (Kochhar and Sechopoulos, 2022). Policy efforts that promote economic stability for these families, such as increasing equal employment opportunities, expanding unemployment insurance eligibility and benefits, providing consistent child care support, and providing frequent regular financial assistance (e.g., the Child Tax Credit monthly payments) are still urgently needed to address the severe issues of financial hardship and economic instability facing these families.

Second, households of Black and Latino(a) background were facing disproportionally more challenges during the pandemic because of structural, systemic racism and discrimination (Iruka et al., 2021). These structural inequalities are rooted in histories of race/ethnicity-based economic exploitation, slavery, segregation (Hannah-Jones, 2020), early colonialism (Eckler, 2020), and anti-immigrant prejudice and policies (Peña-Sullivan, 2020), which cannot—and should not—be left for families of colors themselves to survive traumatic socio-historical events without societal efforts for systemic changes. While acknowledging the perceived positive changes in these families that signaled remarkable resilience, this knowledge should not weaken policy and program efforts that aim to promote racial/ethnic equity and reduce financial disparities. These efforts need to apply a racial/ethnic equity lens to all stages of policy development, such as ensuring meaningful participation and leadership by individuals and communities of color in policy-making processes, adopting a data-driven approach that reveals disparities, evaluating how policies differentially affect diverse families, addressing cultural and linguistic needs, and investing in resources to expand equitable access to resources and services for families from different racial/ethnic backgrounds (The Center for Law and Social Policy, 2018). Moreover, parents of young children often attain these positive changes through tenacious efforts and perseverance through difficult circumstances, which may have long-term physical and mental health consequences. As the theory of John Henryism (James et al., 1983)^1^ suggested, active coping of prolonged stressors in the face of low socioeconomic resources, racism, and systemic exclusion could get under the skin and take tolls on individuals’ health, accelerating the occurrence of physical (e.g., high blood pressure, hypertension) and mental (e.g., depression, psychiatric comorbidities) health issues (James et al., 1983; Bennett et al., 2004). Even though Black and Latino(a) parents were found to exhibit higher levels of positive changes than White parents during the pandemic, many of which were related to forms community cultural wealth that helped to navigate various forms of racism (Yosso, 2005). Researchers, practitioners, and policymakers should be aware of the potential negative health and well-being consequences of prolonged hardworking, active coping, and orientation towards achievement (Perez et al., 2023), which may further exacerbate the existing racial/ethnic health disparities (Watson et al., 2008). Research is still needed to identify the conditions when post-traumatic positive changes would occur without potentially compromising long-term health outcomes.

### 4.4 Limitations

This study has several limitations. There might be reporting biases due to the utilization of only parent-reported surveys, and the validity and reliability of assessed well-being outcomes may be limited because of the use of shortened scales. For instance, child behavioral problems were measured via two items—fussiness/defiance and fear/anxiety symptoms—selected from CBCL, which had limited validity to represent broader externalizing and internalizing problems. Thus, the current study findings were only established in relation to these two specific symptoms, and future studies that examine broader behavioral problems using the full scale are still needed.

Despite the RAPID project’s extensive efforts to recruit families from diverse racial/ethnic backgrounds, the sample used in this study is a majority-White (74.44%) convenience sample. We had small subsamples of participants from American Indian/Alaska Native, Asian, and Native Hawaiian/Pacific Islander, and multi-racial backgrounds, which did not allow us to investigate their specific, within-group experiences. With Black and Latino(a) families, the moderate subsample sizes allowed us to examine the mean-level differences in comparison to households of White and the other racial/ethnic backgrounds but prevented us from further investigating whether the Aim 2 and Aim 3 models varied by race/ethnicity. Moreover, offering surveys in English and Spanish excluded parents who spoke other languages from participation, further limited the representativeness of study sample. Overall, the current study sample was more sociodemographically privileged than the RAPID full sample and the national population, which limited the generalizability of study findings especially with the promotive and protective effects of perceived changes among families of lower-SES or non-White racial/ethnic backgrounds. The overall higher income- and education-levels of this sample may indicate access to additional resources that could confound the effects of perceived changes on the associations between material hardship, parent well-being, and child behaviors.

Another factor that may limit this study’s generalizability was related to participants’ digital capacity. The nature of online surveys might prevent families who did not have access to the Internet or digital equipment from participating this study, so digital inequality could affect the representativeness of the current sample. Lastly, the differences of assessment times between study variables (perceived changes, parent well-being, and child well-being) were fairly short, suggesting that the discovered associations could be transient. Studies are still needed to examine how these five domains of perceived changes could affect parents’ and young children’s emotional well-being in the long term.

## 5 Conclusion

For the whole population of U.S. households with young children, the COVID-19 pandemic caused profound disruptions and severe stress; families of lower-SES and diverse racial/ethnic (especially Black and Latino[a]) background were negatively affected as disproportional rates as a result of prolonged structural inequities. Yet, even in the face of such difficult circumstances, some parents of young children, particularly those from marginalized racial/ethnic backgrounds, exhibited remarkable tenacity, persevered through challenges, and as shown in this study, perceived positive changes in the domains of new possibilities, personal strengths, improved relationships, spiritual growth, and appreciation of life. These perceived positive changes could further enhance the families’ well-being and contribute to the family resilience processes. In this study, we found that after exposure to 2 years of pandemic disruptions, half of parents with young children in the U.S. perceived moderate-to-large degrees of changes across the five domains. Black and Latino(a) parents, in particular, exhibited higher degrees of changes across all five domains than White parents. Parents’ perceived changes functioned in the family resilience processes through its cascading effects on parents’ emotional well-being and subsequently child behaviors. In particular, the change domain of improved relationships could reduce child behavioral problems via reducing parent emotional distress. The change domains of appreciation of life, new possibilities, and personal strengths could buffer the negative “chain of hardship,” particularly the impact of average hardship levels, on parent and child well-being. With 2 years of continuous exposure to pandemic stressors, parents’ perceived changes may reflect their constructive positive changes over time. This lens could be adopted by therapeutic and intervention practices to help families with young children cope with residual challenges from the COVID-19 pandemic, as well as in the face of future similar disruptive events.

## Data availability statement

The raw data supporting the conclusions of this article will be made available by the authors, without undue reservation.

## Ethics statement

The studies involving humans were approved by Institutional Review Board, Stanford University and Institutional Review Board, University of Oregon. The studies were conducted in accordance with the local legislation and institutional requirements. The participants provided their written informed consent to participate in this study.

## Author contributions

SL: Conceptualization, Formal analysis, Writing – original draft. SC: Conceptualization, Resources, Writing – review & editing. JS: Conceptualization, Resources, Writing – review & editing. PF: Conceptualization, Funding acquisition, Methodology, Supervision, Writing – review & editing.

## Footnotes

^1^John Henry was a Black folk hero who worked vigorously enough to compete successfully with a steam powered machine but died as a result of his effort. John Henryism is a hypothesis proposed by Dr. Sherman James that acknowledges the health costs of overcoming economic hardships and racial discrimination. Dr. James proposed this hypothesis while investigating racial health disparities between Black people and other racial groups in North Carolina, where he interviewed a Black man, John Henry Martin, whose situations were evocative of the folk hero John Henry.
