# Supplementary material for: The promotive and protective effects of parents’ perceived changes during the COVID-19 pandemic on emotional well-being among U.S. households with young children: an investigation of family resilience processes
Source: Front Psychol. 2024 Jan 8;14:1270514. doi: 10.3389/fpsyg.2023.1270514 (PMC10800496; doi:10.3389/fpsyg.2023.1270514)
Supplement: Supplementary file 1 [file Table_1.DOCX]

**Supplemental Materials**

Supplemental Table 1. *Comparisons of Demographic Characteristics in the Current Sample, RAPID Full Sample, and National Population*

| Demographic Characteristics | | Current Study Sample  (*N* = 669) | Full RAPID Sample  (*N* = 18,583) | National Population |
| --- | --- | --- | --- | --- |
| Race/ Ethnicity | American Indian/Alaska Native | 0.60% (*n* = 4) | 1.31% (*n* = 244) | 0.8% |
|  | Asian | 4.63% (*n* = 31) | 3.85% (*n* = 715) | 4.6% |
|  | Black | 8.07% (*n* = 54) | 9.80% (*n* = 1,819) | 9.1% |
|  | Latino(a) | 9.57% (*n* = 64) | 14.01% (*n* = 2,600) | 17.6% |
|  | Native Hawaiian/Pacific Islander | 0.15% (*n* = 1) | 0.62% (*n* = 116) | 0.1% |
|  | Non-Latino(a) White | 74.44% (*n* = 498) | 66.62% (*n* = 12,366) | 63.7% |
|  | Multi-race | 1.35% (*n* = 9) | 2.85% (*n* = 529) | 1.9% |
|  | Other Racial/Ethnic Backgrounds | 1.20% (*n* = 8) | 0.93% (*n* = 172) | 2.2% |
| Income Levels | Below 200% FPL | 26.36% (*n* = 175) | 31.37% (*n* = 5,134) | 34.3% |
|  | 200%-400% FPL | 34.04% (*n* = 226) | 36.29% (*n* = 5,940) | 65.7% |
|  | At/Above 400% FPL | 39.61% (*n* = 263) | 32.34% (*n* = 5,292) |  |
| Race/  Ethnicity by Income Levels  (Percentage within each racial/ ethnic group presented) | Black - |  |  | -- |
|  | Below 200% FPL | 48.15% (*n* = 26) | 48.34% (*n* = 699) |  |
|  | 200%-400% FPL | 25.93% (*n* = 14) | 36.79% (*n* = 532) |  |
|  | At/Above 400% FPL | 25.93% (*n* = 14) | 16.94% (*n* = 245) |  |
|  | Latino(a) - |  |  |  |
|  | Below 200% FPL | 41.27% (*n* = 26) | 41.84% (*n* = 905) |  |
|  | 200%-400% FPL | 33.33% (*n* = 21) | 36.85% (*n* = 797) |  |
|  | At/Above 400% FPL | 25.40% (*n* = 16) | 21.31% (*n* = 461) |  |
|  | Non-Latino(a) White |  |  |  |
|  | Below 200% FPL | 23.79% (*n* = 118) | 28.26% (*n* = 3,152) |  |
|  | 200%-400% FPL | 35.48% (*n* = 176) | 35.23% (*n* = 3,930) |  |
|  | At/Above 400% FPL | 40.73% (*n* = 202) | 36.51% (*n* = 4,073) |  |
|  | All Other Racial/Ethnic Groups |  |  |  |
|  | Below 200% FPL | 11.76% (*n* = 6) | 24.23% (*n* = 378) |  |
|  | 200%-400% FPL | 37.25% (*n* = 19) | 43.21% (*n* = 674) |  |
|  | At/Above 400% FPL | 50.98% (*n* = 26) | 32.56% (*n* = 508) |  |
| Respondent Gender | Woman/Transgender Woman | 97.75% (*n* = 652) | 88.48% (*n* = 16,417) | -- |
|  | Man/Transgender Man | 1.95% (*n* = 13) | 10.86% (*n* = 2,015) |  |
|  | Non-Binary | 0.00% (*n* = 0) | 0.17% (*n* = 31) |  |
|  | Not Listed | 0.30% (*n* = 2) | 0.26% (*n* = 48) |  |
|  | Do Not Wish to Disclose | 0.00% (*n* = 0) | 0.24% (*n* = 45) |  |
| Respondent Education | High school diploma/GED or below | 5.86% (*n* = 39) | 11.92% (*n* = 1,769) | 27% |
|  | Some College/Associate Degree | 20.60% (*n* = 137) | 31.66% (*n* = 4,697) | 28% |
|  | Bachelor’s Degree | 33.68% (*n* = 224) | 30.14% (*n* = 4,472) | 23% |
|  | Graduate/Professional Degree | 38.20% (*n* = 254) | 24.09% (*n* = 3,574) | 21% |
|  | Other degrees | 1.65% (*n* = 11) | 1.06% (*n* = 157) | -- |
| Geographic Regions | Midwest | 24.89% (*n* = 166) | 23.24% (*n* = 4,312) | 21.6% |
|  | Northeast | 22.94% (*n* = 153) | 16.25% (*n* = 3,016) | 16.2% |
|  | South | 25.49% (*n* = 170) | 33.96% (*n* = 6,301) | 38.1% |
|  | West | 26.69% (*n* = 178) | 26.55% (*n* = 4,927) | 24.2% |

*Note*. National population data regarding race/ethnicity, income levels, and geographic regions were extracted from the American Community Survey (ACS) 5-year estimates, based on households with at least one child under 6 years old. The national population parent education data were extracted from a report from the Institute of Education Sciences (IES; <https://nces.ed.gov/programs/coe/indicator/cce/family-characteristics>).

| Measurement/Subscales | | Full Sample | By Race/Ethnicity | | | |  | By Income Levels | |
| --- | --- | --- | --- | --- | --- | --- | --- | --- | --- |
|  |  |  | Black | Latino(a) | Other Groups | White |  | Below 200% FPL | At/Above 400% FPL |
| Post-Traumatic Growth Inventory (PTGI) | New Possibilities | .80 | .87 | .82 | .74 | .78 |  | .85 | .85 |
|  | Personal Strengths | .85 | .86 | .86 | .86 | .84 |  | .80 | .80 |
|  | Improved Relationships | .87 | .91 | .88 | .89 | .86 |  | .87 | .87 |
|  | Spiritual Growth | .86 | .89 | .84 | .76 | .85 |  | .82 | .88 |
|  | Appreciation of Life | .77 | .74 | .81 | .64 | .78 |  | .78 | .79 |
| Parent Emotional Distress | April 2022 Timepoint | .85 | .87 | .88 | .88 | .84 |  | .83 | .87 |
|  | F1 Timepoint | .87 | .85 | .88 | .86 | .87 |  | .89 | .86 |

Supplemental Table 2. *Measurement Internal Consistency in the Full and Sub-Samples*.

*Note.* Cronbach’s alpha scores are presented in this table. “Other Groups” indicate all the other racial/ethnic groups combined.

Supplemental Table 3. *SEM Model Examining the Promotive Indirect Effects of Perceived Change Domains on Decreases in Child Behavioral Problems Through Reducing Parent Emotional Distress*

| Paths | *B* (*S.E.*) | β | 95%*CI* of *B* |
| --- | --- | --- | --- |
| *Path a* |  |  |  |
| New Possibilities (April22) → Parent Emotional Distress (F1) | 1.561 (.935) | .079 | [-.272, 3.394] |
| Personal Strengths (April22) → Parent Emotional Distress (F1) | -.302 (.890) | -.017 | [-2.047, 1.443] |
| Improved Relationships (April22) → Parent Emotional Distress (F1) | -2.109 (.904) | -.102 | [-3.881, -.337]* |
| Spiritual Growth (April22) → Parent Emotional Distress (F1) | .028 (.553) | .002 | [-1.056, 1.111] |
| Appreciation of Life (April22) → Parent Emotional Distress (F1) | 1.103 (.771) | .061 | [-.408, 2.614] |
| *Path b* |  |  |  |
| Parent Emotional Distress (F1) → Child Fussiness/Defiance (F2) | .004 (.001) | .171 | [.002, .007]** |
| Parent Emotional Distress (F1) → Child Fear/Anxiety (F2) | .004 (.001) | .165 | [.002, .006]** |
| *Path c’* |  |  |  |
| New Possibilities (April22) → Child Fussiness/Defiance (F2) | -.053 (.042) | -.109 | [-.135, .030] |
| Personal Strengths (April22) → Child Fussiness/Defiance (F2) | -.015 (.039) | -.034 | [-.090, .061] |
| Improved Relationships (April22) → Child Fussiness/Defiance (F2) | -.012 (.043) | -.024 | [-.096, .071] |
| Spiritual Growth (April22) → Child Fussiness/Defiance (F2) | .005 (.024) | .012 | [-.041, .051] |
| Appreciation of Life (April22) → Child Fussiness/Defiance (F2) | .064 (.035) | .144 | [-.004, .131] |
| New Possibilities (April22) → Child Fear/Anxiety (F2) | -.054 (.039) | -.114 | [-.131, .023] |
| Personal Strengths (April22) → Child Fear/Anxiety (F2) | .007 (.036) | .017 | [-.063, .078] |
| Improved Relationships (April22) → Child Fear/Anxiety (F2) | .039 (.040) | .078 | [-.039, .117] |
| Spiritual Growth (April22) → Child Fear/Anxiety (F2) | -.001 (.022) | -.004 | [-.045, .042] |
| Appreciation of Life (April22) → Child Fear/Anxiety (F2) | .036 (.032) | .084 | [-.027, .100] |
| *Covariates* |  |  |  |
| Parent Emotional Distress (April22) → Parent Emotional Distress (F1) | .755 (.029) | .745 | [.698, .813]*** |
| Black → Parent Emotional Distress (F1) | -.853 (2.321) | -.010 | [-5.384, 3.715] |
| Latino(a) → Parent Emotional Distress (F1) | .753 (2.132) | .010 | [-3.425, 4.931] |
| Other minorities → Parent Emotional Distress (F1) | -.906 (2.351) | -.011 | [-5.514, 3.703] |
| Poverty → Parent Emotional Distress (F1) | 2.394 (1.560) | .046 | [-.664, 5.452] |
| Child Fussiness/Defiance (F1) → Child Fussiness/Defiance (F2) | .365 (.050) | .386 | [.268, .462]*** |
| Black → Child Fussiness/Defiance (F2) | -.055 (.089) | -.026 | [-.229, .119] |
| Latino(a) → Child Fussiness/Defiance (F2) | .124 (.084) | .064 | [-.041, .288] |
| Other minorities → Child Fussiness/Defiance (F2) | -.085 (.105) | -.041 | [-.292, .122] |
| Poverty → Child Fussiness/Defiance (F2) | .029 (.062) | .023 | [-.093, .151] |
| Child Fear/Anxiety (F1) → Child Fear/Anxiety (F2) | .485 (.052) | .458 | [.383, .588]*** |
| Black → Child Fear/Anxiety (F2) | .008 (.083) | .004 | [-.154, .170] |
| Latino(a) → Child Fear/Anxiety (F2) | .134 (.079) | .071 | [-.021, .288] |
| Other minorities → Child Fear/Anxiety (F2) | -.050 (.098) | -.024 | [-.243, .143] |
| Poverty → Child Fear/Anxiety (F2) | .028 (.058) | .022 | [-.085, .142] |
| *Model Fit Indices* | χ^2^(36) = 97.001 (*p* < .001), CFI = .917, SRMR = .041 | | |

*Note.* April22 = survey administered during April 2022, when PTGI items were assessed; F1 = each participant’s first response after the April 2022 assessment; F2 = each participant’s second response after the April 2022 assessment. Black, Latino(a), Other Minority variables were all 0/1 binary variables. Poverty was also a binary variable, with “1” indicating below 200% FPL. **p* < .05, ***p* < .01, *** *p* < .001.

Supplemental Table 4. *SEM Model Examining the “Chain of Hardship”*

| Paths | *B* (*S.E.*) | β | 95%*CI* of *B* |
| --- | --- | --- | --- |
| *Path a* |  |  |  |
| Material Hardship Mean Level (At/Pre April22) → Parent Emotional Distress (F1) | 1.928 (.762) | .088 | [.434, 3.422]* |
| Material Hardship Unpredictability (At/Pre April22) → Parent Emotional Distress (F1) | 1.508 (.689) | .066 | [.157, 2.859]* |
| *Path b* |  |  |  |
| Parent Emotional Distress (F1) → Child Fussiness/Defiance (F2) | .003 (.001) | .133 | [.001, .006]* |
| Parent Emotional Distress (F1) → Child Fear/Anxiety (F2) | .004 (.001) | .146 | [.001, .006]** |
| *Path c’* |  |  |  |
| Material Hardship Mean Level (At/Pre April22) → Child Fussiness/Defiance (F2) | .078 (.029) | .143 | [.021, .134]** |
| Material Hardship Unpredictability (At/Pre April22) → Child Fussiness/Defiance (F2) | .100 (.028) | .178 | [.045, .154]*** |
| Material Hardship Mean Level (At/Pre April22) → Child Fear/Anxiety (F2) | .004 (.028) | .008 | [-.050, .059] |
| Material Hardship Unpredictability (At/Pre April22) → Child Fear/Anxiety (F2) | .054 (.027) | .099 | [.002, .106]* |
| *Covariates* |  |  |  |
| Parent Emotional Distress (April22) → Parent Emotional Distress (F1) | 7.013 (.326) | .683 | [6.374, 7.651]*** |
| Black → Parent Emotional Distress (F1) | 1.303 (2.476) | .015 | [-3.550, 6.155] |
| Latino(a) → Parent Emotional Distress (F1) | 2.555 (2.285) | .032 | [-1.922, 7.033] |
| Other minorities → Parent Emotional Distress (F1) | .003 (2.466) | .000 | [-4.830, 4.835] |
| Poverty → Parent Emotional Distress (F1) | 1.615 (1.728) | .031 | [-1.773, 5.002] |
| Child Fussiness/Defiance (F1) → Child Fussiness/Defiance (F2) | .368 (.048) | .386 | [.274, .463]*** |
| Black → Child Fussiness/Defiance (F2) | -.057 (.084) | -.027 | [-.221, .107] |
| Latino(a) → Child Fussiness/Defiance (F2) | .067 (.080) | .035 | [-.090, .225] |
| Other minorities → Child Fussiness/Defiance (F2) | -.104 (.103) | -.049 | [-.305, .097] |
| Poverty → Child Fussiness/Defiance (F2) | -.079 (.065) | -.061 | [-.206, .048] |
| Child Fear/Anxiety (F1) → Child Fear/Anxiety (F2) | .495 (.052) | .465 | [.392, .598]*** |
| Black → Child Fear/Anxiety (F2) | .033 (.080) | .016 | [-.124, .189] |
| Latino(a) → Child Fear/Anxiety (F2) | .118 (.078) | .062 | [-.035, .270] |
| Other minorities → Child Fear/Anxiety (F2) | -.034 (.098) | -.017 | [-.226, .158] |
| Poverty → Child Fear/Anxiety (F2) | -.014 (.062) | -.011 | [-.135, .107] |
| *Model Fit Indices* | χ^2^(36) = 44.154 (*p* < .001), CFI = .955, SRMR = .029 | | |

*Note.* April22 = survey administered during April 2022, when PTGI items were assessed; F1 = each participant’s first response after the April 2022 assessment; F2 = each participant’s second response after the April 2022 assessment. Black, Latino(a), Other Minority variables were all 0/1 binary variables. Poverty was also a binary variable, with “1” indicating below 200% FPL. **p* < .05, ***p* < .01, *** *p* < .001.

Supplemental Table 5. *SEM Models Examining the Protective Effects of Perceived Change Domains on the “Chain of Hardship”*

| Paths | New Possibilities | | | Personal Strengths | | | Improved Relationships | | | Spiritual Growth | | | Appreciation of Life | | |
| --- | --- | --- | --- | --- | --- | --- | --- | --- | --- | --- | --- | --- | --- | --- | --- |
|  | *B* (*S.E.*) | β | 95%*CI* of *B* | *B* (*S.E.*) | β | 95%*CI* of *B* | *B* (*S.E.*) | β | 95%*CI* of *B* | *B* (*S.E.*) | β | 95%*CI* of *B* | *B* (*S.E.*) | β | 95%*CI* of *B* |
| *Path a* |  |  |  |  |  |  |  |  |  |  |  |  |  |  |  |
| HS-Level → P-Distress | 5.676 (1.930) | .258 | [2.501, 9.460]** | 6.165 (2.090) | .280 | [2.069, 10.262]** | 4.250 (1.740) | .193 | [.839, 7.661]* | 2.812 (1.076) | .128 | [.703, 4.921]** | 9.051 (2.497) | .412 | [4.158, 13.945]*** |
| HS-Unpdb. → P-Distress | -.707 (1.893) | -.031 | [-4.418, 3.004] | .073 (1.997) | .003 | [-3.841, 3.987] | -1.661 (1.971) | -.073 | [-5.523, 2.201] | 1.239 (.909) | .065 | [-.544, 3.021] | .771 (2.179) | .034 | [-3.499, 5.041] |
| PTGI-D → P-Distress | 1.737 (1.809) | .039 | [-1.808, 5.283] | .414 (1.706) | .010 | [-2.929, 3.757] | -.895 (1.850) | -.020 | [-4.540, 2.749] | .083 (1.312) | .003 | [-2.489, 2.655] | 4.166 (1.749) | .092 | [.739, 7.594]* |
| HS-Level* PTGI-D → P-Distress | -2.813 (1.311) | -.189 | [-5.383, -.244]* | -2.781 (1.298) | -.202 | [-5.325, -.237]* | -1.867 (1.277) | -.116 | [-4.371, .636] | -.967 (.863) | -.055 | [-2.658, .725] | -4.437 (1.453) | -.346 | [-7.285, -1.590]** |
| HS-Unpdb*PTGI-D → P-Distress | 1.777 (1.459) | .104 | [-1.083, 4.637] | 1.048 (1.397) | .067 | [-1.689, 3.786] | 2.652 (1.536) | .150 | [-.359, 5.662] | .399 (.871) | .020 | [-1.309, 2.107] | .352 (1.356) | .025 | [-2.307, 3.010] |
| *Path b* |  |  |  |  |  |  |  |  |  |  |  |  |  |  |  |
| P-Distress → C-Fussy | .004 (.001) | .145 | [.001, .006]** | .003 (.001) | .135 | [.001, .006]** | .003 (.001) | .136 | [.001, .006]** | .003 (.001) | .139 | [.001, .006]** | .004 (.001) | .146 | [.001, .006]** |
| P-Distress → C-Fear | .003 (.001) | .135 | [.001, .006]** | .003 (.001) | .135 | [.001, .006]** | .004 (.001) | .153 | [.001, .006]** | .004 (.001) | .159 | [.001, .006]** | .003 (.001) | .132 | [.001, .006]** |
| *Path c’* |  |  |  |  |  |  |  |  |  |  |  |  |  |  |  |
| HS-Level → C-Fussy | -.040 (.070) | -.073 | [-.178, .098] | .020 (.079) | .037 | [-.136, .176] | .041 (.067) | .075 | [-.090, .171] | .045 (.040) | .082 | [-.034, .123] | -.072 (.094) | -.133 | [-.256, .112] |
| HS-Unpdb. → C-Fussy | .157 (.075) | .280 | [.011, .304]* | .122 (.076) | .217 | [-.028, .271] | .226 (.076) | .401 | [.077, .374]** | .111 (.035) | .197 | [.042, .180]** | .117 (.078) | .207 | [-.037, .270] |
| PTGI-D → C-Fussy | -.119 (.072) | -.107 | [-.260, .023] | -.048 (.067) | -.046 | [-.180, .084] | -.002 (.076) | .002 | [-.151, .147] | -.011 (.050) | -.015 | [-.109, .087] | -.015 (.069) | -.014 | [-.151, .120] |
| HS-Level* PTGI-D → C-Fussy | .093 (.049) | .254 | [-.004, .190] | .041 (.051) | .121 | [-.059, .141] | .032 (.052) | .081 | [-.069, .134] | .041 (.034) | .093 | [-.027, .108] | .092 (.056) | .291 | [-.017, .201] |
| HS-Unpdb*PTGI-D → C-Fussy | -.048 (.058) | -.114 | [-.162, .066] | -.016 (.054) | -.042 | [-.122, .089] | -.105 (.059) | -.241 | [-.221, .011] | -.021 (.035) | -.042 | [-.090, .048] | -.012 (.050) | -.033 | [-.110, .087] |
| HS-Level → C-Fear | .077 (.067) | .146 | [-.055, .209] | .102 (.075) | .194 | [-.044, .249] | .040 (.064) | .075 | [-.085, .164] | -.030 (.038) | -.056 | [-.105, .045] | .079 (.089) | .149 | [-.096, .253] |
| HS-Unpdb. → C-Fear | .158 (.071) | .289 | [.018, .298]* | .143 (.072) | .261 | [.001, .284]* | .141 (.073) | .257 | [-.001, .283] | .047 (.033) | .086 | [-.018, .113] | .078 (.074) | .143 | [-.067, .224] |
| PTGI-D → C- Fear | .104 (.069) | .097 | [-.032, .240] | .150 (.064) | .147 | [.025, .274]* | .146 (.073) | .133 | [.003, .289]* | .001 (.047) | .001 | [-.092, .093] | .144 (.066) | .132 | [.015, .274]* |
| HS-Level* PTGI-D → C- Fear | -.056 (.047) | -.158 | [-.149, .037] | -.071 (.048) | -.215 | [-.165, .023] | -.030 (.049) | -.077 | [-.127, .067] | .041 (.033) | .097 | [-.023, .106] | -.049 (.053) | -.159 | [-.152, .054] |
| HS-Unpdb*PTGI-D → C- Fear | -.087 (.056) | -.212 | [-.195, .022] | -.068 (.051) | -.179 | [-.168, .032] | -.074 (.057) | -.175 | [-.185, .037] | .009 (.033) | .019 | [-.057, .074] | -.018 (.048) | -.053 | [-.111, .076] |
| *Covariates* |  |  |  |  |  |  |  |  |  |  |  |  |  |  |  |
| P-Distress (Apr22)  → P-Distress (F1) | 6.971 (.326) | .679 | [6.333, 7.609]  *** | 6.925 (.327) | .675 | [6.283, 7.567]  *** | 6.996 (.325) | .682 | [6.358, 7.634]  *** | 6.974 (.327) | .679 | [6.333, 7.615]  *** | 6.903 (.325) | .672 | [6.266, 7.540]  *** |
| Black → P-Distress | .840 (2.485) | .010 | [-4.030, .5.710] | 1.401 (2.481) | .016 | [-3.462, 6.265] | 1.475 (2.494) | .017 | [-3.413, 6.362] | 1.491 (2.513) | .017 | [-3.435, 6.417] | .164 (2.485) | .002 | [-4.797, 5.036] |
| Latino(a) → P-Distress | 2.093 (2.308) | .027 | [-2.430, 6.616] | 2.519 (2.283) | .032 | [-1.957, 6.994] | 2.744 (2.295) | .035 | [-1.753, 7.242] | 2.737 (2.297) | .035 | [-1.765, 7.238] | 2.356 (2.280) | .030 | [-2.113, 6.824] |
| Other → P-Distress | -.439 (2.464) | -.005 | [-5.269, 4.391] | -.242 (2.464) | -.003 | [-5.072, 4.588] | -.285 (2.457) | -.003 | [-5.102, 4.531] | -.092 (2.463) | -.001 | [-4.918, 4.735] | -.895 (2.461) | -.010 | [-5.719, 3.930] |
| Poverty → P-Distress | 1.439 (1.735) | .027 | [-1.961, 4.839] | 1.228 (1.730) | .023 | [-2.163, 4.620] | 1.296 (1.753) | .025 | [-2.139, 4.731] | 1.357 (1.741) | .026 | [-2.055, 4.769] | 1.817 (1.728) | .034 | [-1.570, 5.203] |
| C-Fussy (F1) → C-Fussy (F2) | .374 (.048) | .391 | [.279, .468]*** | .376 (.049) | .393 | [.280, .471]*** | .369 (.048) | .385 | [.274, .463]*** | .369 (.049) | .386 | [.273, .464]*** | .379 (.048) | .396 | [.284, .474]*** |
| Black → C-Fussy | 0.028 (.084) | -.013 | [-.193, .137] | -.051 (.084) | -.024 | [-.216, .114] | -.044 (.085) | -.021 | [-.210, .122] | -.063 (.085) | -.030 | [-.230, .103] | -.052 (.085) | -.025 | [-.218, .114] |
| Latino(a) → C-Fussy | .102 (.082) | .053 | [-.059, .263] | .077 (.081) | .040 | [-.082, .236] | .083 (.081) | .043 | [-.077, .242] | .070 (.081) | .036 | [-.088, .229] | .055 (.081) | .028 | [0.104, .215] |
| Other → C-Fussy | -.080 (.102) | -.038 | [-.280, .121] | -.095 (.103) | -.045 | [-.296, .106] | -.086 (.193) | -.041 | [-.287, .115] | -.091 (.103) | -.043 | [-.293, .110] | -.091 (.103) | -.043 | [-.292, .111] |
| Poverty → C-Fussy | -.085 (.064) | -.065 | [-.211, .041] | -.081 (.065) | -.062 | [-.208, .046] | -.084 (.065) | -.065 | [-.211, .044] | -.067 (.065) | -.052 | [-.195, .061] | -.076 (.065) | -.058 | [-.203, .052] |
| C-Fear (F1) → C-Fear (F2) | .492 (.052) | .464 | [.390, .594]*** | .496 (.052) | .467 | [.394, .597]*** | .488 (.052) | .458 | [.385, .591]*** | .487 (.053) | .458 | [.385, .590]*** | .499 (.052) | .469 | [.397, .601]*** |
| Black → C-Fear | .018 (.080) | .009 | [-.140, .175] | .007 (.080) | .004 | [-.149, .163] | .009 (.081) | .004 | [-.150, .167] | .017 (.081) | .008 | [-.142, .175] | -.003 (.081) | -.001 | [-.161, .155] |
| Latino(a) → C-Fear | .131 (.079) | .069 | [-.025, .287] | .113 (.078) | .060 | [-.040, .265] | .105 (.078) | .055 | [-.049, .259] | .110 (.078) | .058 | [-.043, .263] | .099 (.078) | .052 | [-.054, .253] |
| Other → C-Fear | -.033 (.098) | -.016 | [-.225, .158] | -.041 (.097) | -.020 | [-.231, .150] | -.041 (.098) | -.020 | [-.232, .151] | -.023 (.098) | -.011 | [-.215, .169] | -.059 (.098) | -.029 | [-.251, .132] |
| Poverty → C-Fear | -.015 (.062) | -.012 | [-.136, .106] | -.009 (.061) | -.007 | [-.129, .111] | -.007 (.062) | -.006 | [-.129, .115] | -.005 (.062) | -.004 | [-.127, .117] | .001 (.062) | .000 | [-.121, .122] |
| *Model Fit Indices* | χ^2^(15) = 48.759 (*p* < .001), CFI = .953, SRMR = .024 | | | χ^2^(15) = 45.989 (*p* < .001), CFI = .956, SRMR = .023 | | | χ^2^(36) = 46.689 (*p* < .001), CFI = .955, SRMR = .023 | | | χ^2^(36) = 45.302 (*p* < .001), CFI = .957, SRMR = .024 | | | χ^2^(36) = 47.653 (*p* < .001), CFI = .954, SRMR = .023 | | |

*Note.* HS-Level = Material hardship mean level, HS-Unpdb. = Material hardship unpredictability, P-Distress = Parent emotional distress, PTGI-D = Examined PTGI Domain, as indicated by the top label in each column section, C-Fussy = Child fussiness/defiance, C-Fear = Child fear/anxiety, Other = Other minorities. Apr22 = survey administered during April 2022, when PTGI items were assessed; F1 = each participant’s first response after the April 2022 assessment; F2 = each participant’s second response after the April 2022 assessment. Black, Latino(a), Other Minority variables were all 0/1 binary variables. Poverty was also a binary variable, with “1” indicating below 200% FPL. **p* < .05, ***p* < .01, *** *p* < .001.
